# Non-transgenic, PAMAM co-delivery DNA of interactive proteins NbCRVP and NbCalB endows *Nicotiana benthamiana* with a stronger antiviral effect to RNA viruses

**DOI:** 10.1186/s12951-023-02252-z

**Published:** 2024-01-08

**Authors:** Liyun Song, Daoshun Zhang, Tianbo Liu, Changqing Jiang, Bin Li, Changquan Li, Lili Shen, Ying Li, Fenglong Wang, Yubing Jiao, Jinguang Yang

**Affiliations:** 1 Key Laboratory of Tobacco Pest Monitoring, Controlling and Integrated Management, Tobacco Research Institute of Chinese Academy of Agricultural Sciences, Qingdao, 266101 China; 2 College of Agriculture and Forestry Science and Technology, Weifang Vocational College, Weifang, 262737 China; 3 Institute of Agricultural Resources and Regional Planning, Chinese Academy of Agricultural Sciences, Beijing, 100081 China; 4 Tobacco Research Institute of Hunan Province, Hunan, 410004 China; 5 Hubei Engineering Research Center for Pest Forewarning and Management, Yangtze University, Jingzhou, 434025 China; 6 Sichuan Tobacco Company, Chengdu, 610000 China; 7 Liupanshui City Company of Guizhou Tobacco Company, Liupanshui, 553000 Guizhou China

**Keywords:** Cysteine-rich venom protein, Calcium-dependent lipid-binding (CaLB domain) family protein, Poly(amidoamine) (PAMAM), PAMAM@NbCRVP:NbCalB, Broad-spectrum antiviral effect

## Abstract

**Background:**

Viral diseases continue to pose a major threat to the world’s commercial crops. The in-depth exploration and efficient utilization of resistance proteins have become crucial strategies for their control. However, current delivery methods for introducing foreign DNA suffer from host range limitations, low transformation efficiencies, tissue damage, or unavoidable DNA integration into the host genome. The nanocarriers provides a convenient channel for the DNA delivery and functional utilization of disease-resistant proteins.

**Results:**

In this research, we identified a cysteine-rich venom protein (NbCRVP) in *Nicotiana benthamiana* for the first time. Virus-induced gene silencing and transient overexpression clarified that NbCRVP could inhibit the infection of tobacco mosaic virus, potato virus Y, and cucumber mosaic virus, making it a broad-spectrum antiviral protein. Yeast two-hybrid assay, co-immunoprecipitation, and bimolecular fluorescence complementation revealed that calcium-dependent lipid-binding (CaLB domain) family protein (NbCalB) interacted with NbCRVP to assist NbCRVP playing a stronger antiviral effect. Here, we demonstrated for the first time the efficient co-delivery of DNA expressing NbCRVP and NbCalB into plants using poly(amidoamine) (PAMAM) nanocarriers, achieving stronger broad-spectrum antiviral effects.

**Conclusions:**

Our work presents a tool for species-independent transfer of two interacting protein DNA into plant cells in a specific ratio for enhanced antiviral effect without transgenic integration, which further demonstrated new strategies for nanocarrier-mediated DNA delivery of disease-resistant proteins.

**Graphical abstract:**

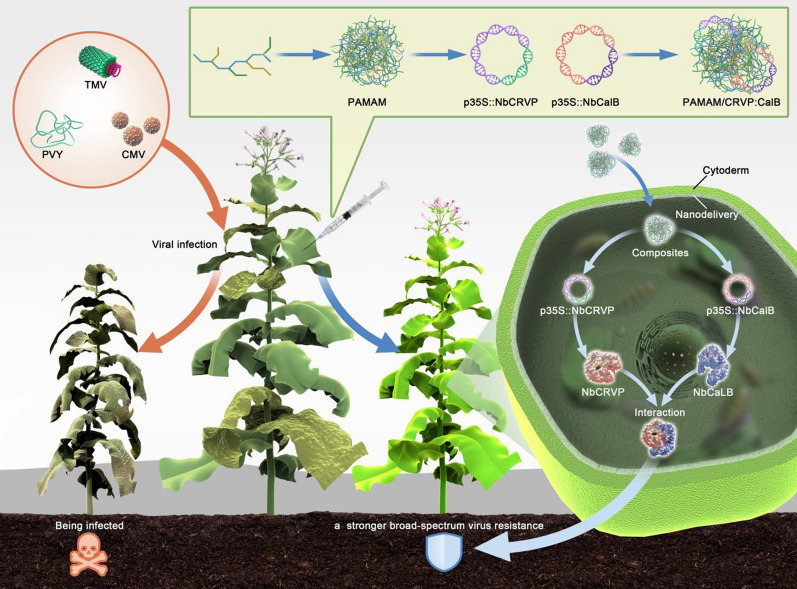

**Supplementary Information:**

The online version contains supplementary material available at 10.1186/s12951-023-02252-z.

## Background

The discovery and functional elucidation of plant disease-resistant genes not only provide an experimental basis for revealing the mechanism of plant immune regulation but also lay a theoretical foundation for efficient improvement of crop quality. However, conventional transgenic strategies mediated by *Agrobacterium tumefaciens* have disadvantages such as a long cycle, limited host range, and inevitable DNA integration to plant genomes. Nanocarrier-based molecular delivery is a promising technique for systematically improving the quality and characteristics of commercial crops. Various metals, nonmetals, and polymer-based nanocomposites can be combined with bioactive molecules to serve as gene delivery nanocarriers. Studies have shown that these biomolecule/nanocarrier complexes can translocate across plant cell walls and into the cytoplasm [1]. After foliar spraying, biomolecule/nanocarrier conjugates can be transferred into plant cells through leaf cuticle or stomatal pathways [2–5]. Furthermore, nanocarriers have also been used to protect these biomolecules from various degradation processes in plant cells [6]. For example, *N*-2-hydroxypropyl trimethyl ammonium chloride chitosan can protect the plasmid carrying the resistance protein NbMLP28 from nuclease degradation and successfully delivery it to achieve the antiviral effect [7].

Cysteine-rich secreted protein (CRISP) is a single-chain bioactive polypeptide with a molecular weight of 20–30 kDa, which is present in snake venom, reptile venom ducts [8–10], salivary glands, pancreatic tissue, and reproductive tract [11–15]. CRISP belongs to the CRISP, antigen 5, and pathogenesis-related 1 protein (CAP) superfamily of proteins [16]. Some snake venom CRISPs have been tested for biological activity in crickets and cockroaches [17]. Snake venom CRISP has been shown to block the activity of L-type Ca^2+^ and/or K^+^ channels and cyclic nucleotide-gated ion channels, thereby preventing smooth muscle cell contraction [10, 18–22]. For example, CRISP family proteins catrin, piscivorin, and ophanin from the snake *Crotalus atrox* caused moderate blockage of L-type calcium channels and partially inhibited the contraction of smooth muscle fibers of the mouse tail artery [10]. Although most of the biological targets of snake venom CRISPs described to date are ion channels [18–23], their functional and molecular targets remain to be determined.

Cytosolic phospholipase A2, phospholipase C (PLC), calmodulin, and calcium-dependent calmodulin-independent protein kinase possess Ca^2+^-binding domains, including a Ca^2+^-dependent lipid-binding (CaLB) domain or a C2 domain. Many C2 domains bind to phospholipid membranes in the presence of Ca^2+^ [24]. Reports have described the involvement of C2 domain proteins in plant responses to abiotic and biotic stresses. For instance, *Arabidopsis thaliana* C2 domain protein BAP1 acts as a negative regulator of bio-stimulation-induced programmed cell death [25, 26], and the lipid-binding C2 domain of V3-PLC3 is critical for targeting the protein to the plasma membrane in response to abiotic stress in mung bean [27]. A novel abiotic stress response inhibitor, the calcium-dependent lipid-binding (CalB) protein, is identified in *A. thaliana* [28]. Compared to many animal C2 domain proteins, only a few C2 domain proteins have been reported in plants. However, it is likely that many C2 domain Ca^2+^ sensing proteins are involved in plant stress signal transduction as positive or negative regulators of stress signaling cascades.

**Fig. 1:**
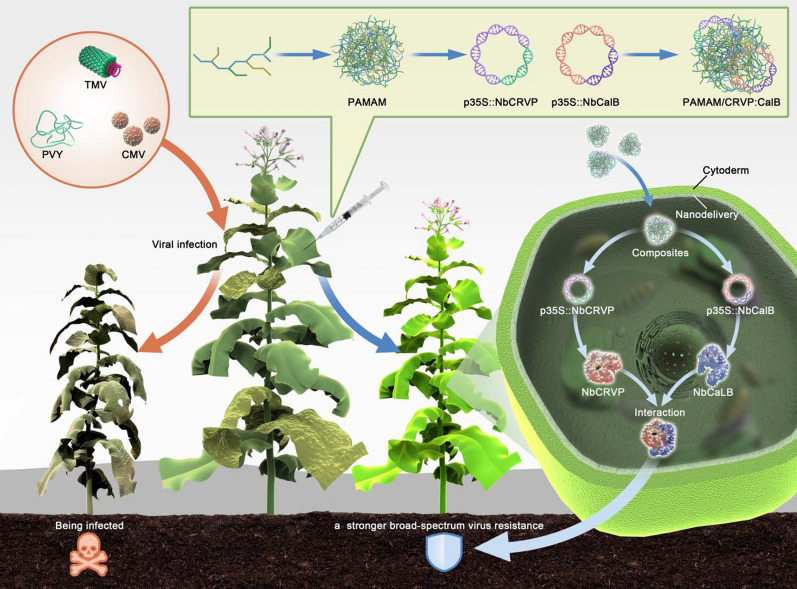
Graphical summary of this study

In this study, we identified a cysteine-rich venom protein (NbCRVP) and its interacting protein calcium-dependent lipid-binding (CaLB domain) family protein (NbCalB) in *Nicotiana benthamiana* for the first time and utilized poly(amidoamine) (PAMAM) as the delivery vehicle, which is a three-dimensional, highly branched dendrimer with monodispersity, low immunogenicity, and low cytotoxicity. PAMAM dendrimers interact with plasmid DNA (pDNA) via electrostatic interactions and condense them to form dendrimer complexes. The dendritic complexes facilitate cellular uptake of DNA, which is eventually released from endosomes after cell entry through the well-known ‘proton sponge effect’ [29]. We successfully prepared PAMAM@NbCRVP:NbCalB complexes to endow NbCRVP with stronger antiviral effect and achieve broad-spectrum antiviral effect to RNA viruses. This study aimed to provide a platform to deliver two or more interacting resistant proteins at different ratios through nanocarriers to achieve enhanced antiviral effects (Fig. 1).

## Results

### Identification and expression pattern of the novel protein NbCRVP

The open reading frame sequence of a cysteine-rich venom protein in *N. benthamiana* was amplified using primers CRVPF/CRVPR (Additional file 2: Table S1) for the first time and named NbCRVP basing on the referenced genome of *N. benthamiana* (https://solgenomics.net/organism/Nicotiana_benthamiana/genome, accessed on May 20, 2020). SMART analysis (http://smart.embl-heidelberg.de/, accessed on February 5, 2020) revealed that the obtained NbCRVP sequence contained an SCP domain, which is the CAP domain shared by all members of the CAP superfamily. Monitoring *NbCRVP* expression under viral infection showed that *NbCRVP* expression was induced at 1 day post-viral inoculation (dpi) and reached its peak at 5 dpi (Additional file 1: Fig. S1a). Moreover, *NbCRVP* expression was detected in roots, stems, leaves, and flowers of the wild-type *N. benthamiana*, with the highest expression level found in the flower tissues (Additional file 1: Fig. S1b). Laser confocal observation of the subcellular localization of NbCRVP showed that the protein was distributed in the entire cell outline, but not in the nucleus. (Additional file 1: Figs. S1e, S2). Spraying 0.5 mM SA, 0.1 mM Me-JA, and 0.05 mM ethephon all upregulated *NbCRVP* expression by 4.15, 4.02, and 9.05 folds, respectively (Additional file 1: Fig. S1c). By contrast, silencing the key genes *NPR1*, *COI1,* and *EIN2* of SA, JA and ET signaling pathways showed only TRV::NPR1 downregulated *NbCRVP* expression, with a 27% reduction (Additional file 1: Fig. S1d). These results suggest that NbCRVP may respond to viral infection.

### Silencing *NbCRVP* up-regulates the sensitivity of *N. benthamiana* to several RNA viruses

**Fig. 2:**
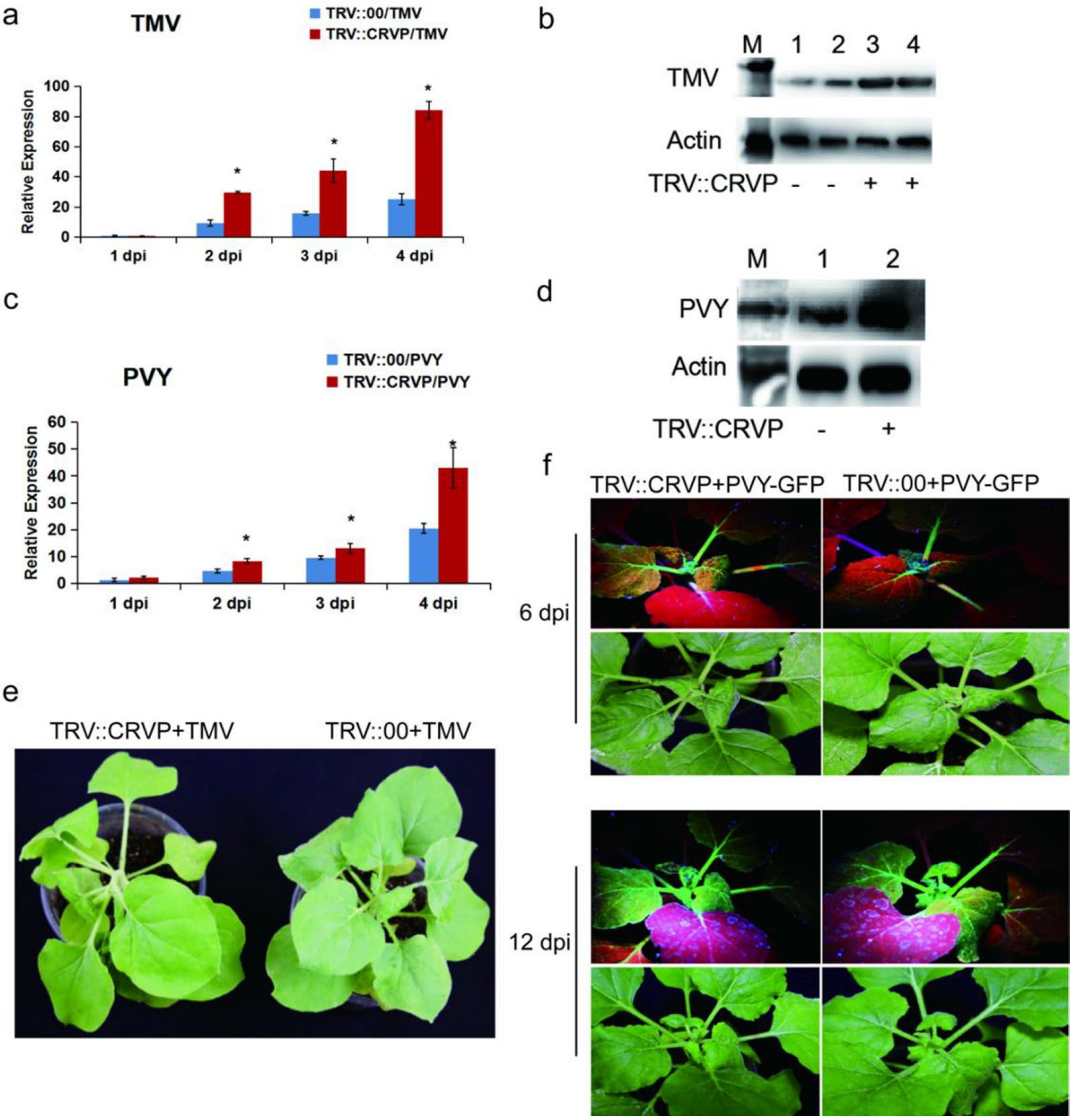
Silencing *NbCRVP* promotes both TMV and PVY infection in *N. benthamiana*. **a** Effect of silencing *NbCRVP* on TMV infection detected by RT-qPCR at 1, 2, 3, and 4 dpi. **b** Effect of silencing *NbCRVP* on TMV infection at the protein level. **c** Effect of silencing *NbCRVP* on PVY infection detected by RT-qPCR at 1, 2, 3, and 4 dpi. **d** Effect of silencing *NbCRVP* on PVY infection at protein levels. **e** Effects of silencing *NbCRVP* on biological symptoms of TMV infection. **f** Effects of silencing *NbCRVP* on GFP signals after PVY-GFP infection. All data were analyzed using the independent sample t test. * indicates significant differences between two different treatments (*P* < 0.05)

To clarify the function of *NbCRVP*, VIGS technology and viral inoculation experiment were used to ascertain its biological function from the expression difference of viral CP at the gene and protein levels. Firstly, the silencing efficiency of *NbCRVP* was detected at 14 days after infiltration of pTRV vectors (Additional file 1: Fig. S3), the virus was innoculated at 15 days and then the effect of silencing *NbCRVP* on viral infection was detected. RT-qPCR data showed that the expression of TMV CP mRNA was significantly higher than that of the control group after silencing *NbCRVP*, which were 2.44 times and 1.46 times at 2 and 3 dpi respectively (Fig. 2a). WB results also confirmed that the TMV CP content in TRV::*CRVP* group was higher than that in the control group (TRV::00) at 3 dpi (Fig. 2b). The biological symptoms of viral infection in the treatment group were also significantly more severe than those in the control group, and the treatment group showed more severe symptoms of necrosis of veins and lodging at 4 dpi (Fig. 2e). All these results confirmed that silencing *NbCRVP* promoted TMV infection to plants.

The PVY-GFP inoculation assay also detected the levels of PVY CP RNA and protein levels in TRV::*CRVP* and TRV::00 groups. At the mRNA level, the *CP* content of the treatment group was 1.78, 1.36, and 2.10 times that of the control group at 2, 3 and 4 dpi, respectively (Fig. 2c). At 4 dpi, the PVY CP antibody imprinted a stronger immune signal in TRV::CRVP samples (Fig. 2d). Moreover, the GFP signal of the treatment group was also stronger than that of the control group at 6 dpi and 12 dpi (Fig. 2f). These results displayed that silencing *NbCRVP* promoted PVY infection to plants. Taken together, silencing *NbCRVP* up-regulated the sensitivity of *N. benthamiana* to several RNA viruses.

### Overexpression of *NbCRVP* enhances the resistance of *N. benthamiana* to RNA viruses

**Fig. 3:**
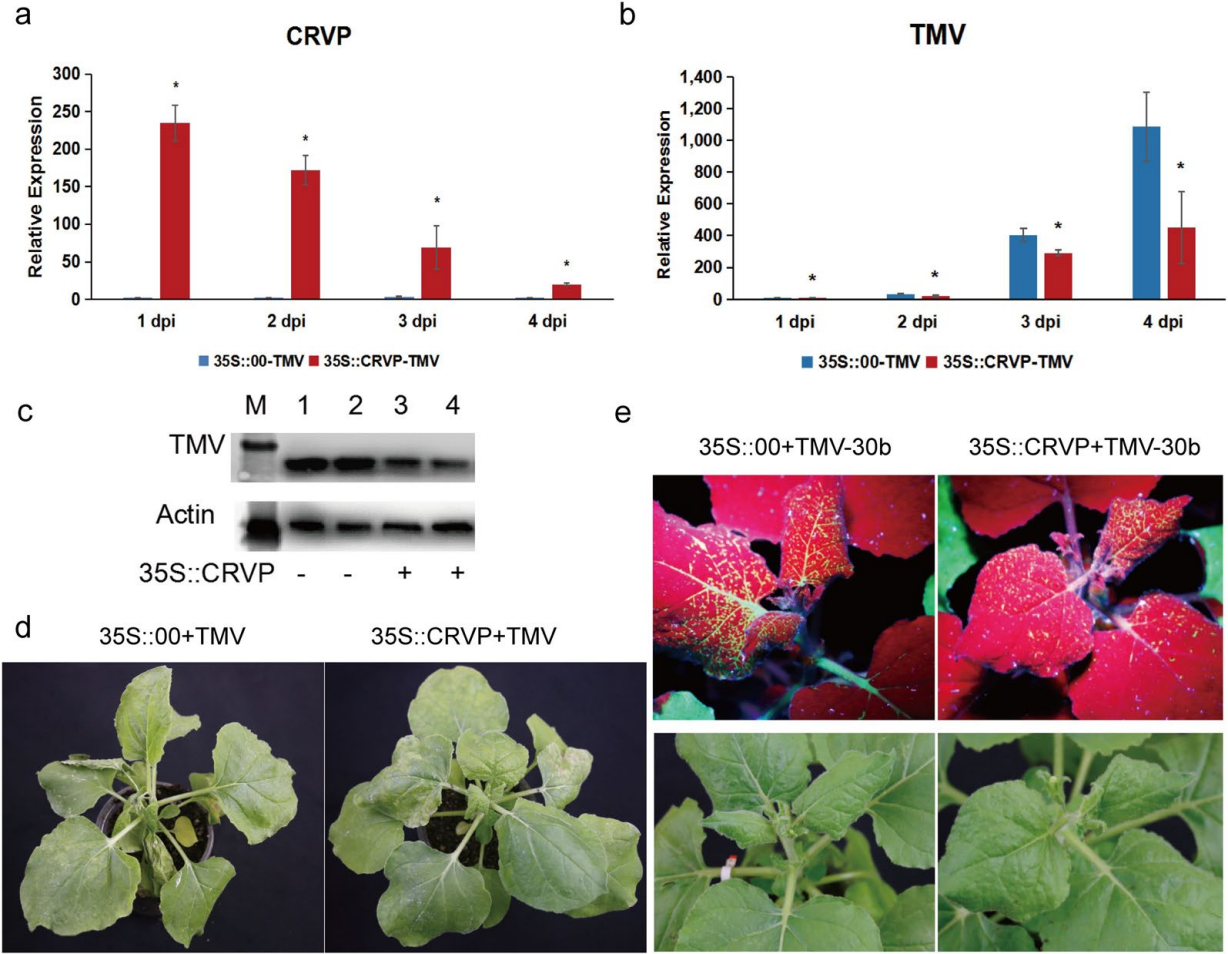
Overexpression of *NbCRVP* inhibits TMV infection to *N. benthamiana*. **a** *NbCRVP* levels after its overexpression*.* **b** TMV CP mRNA levels after *NbCRVP* overexpression at 1, 2, 3, and 4 dpi. **c** Effect of *NbCRVP* overexpression on TMV infection at the protein level. **d** Effect of *NbCRVP* overexpression on biological symptoms of TMV infection. **e** Effect of *NbCRVP* overexpression on GFP signals after TMV-30b infection. The data were analyzed using the independent sample t test, and * indicates significant differences between the two different treatments (*P* < 0.05)

To further confirm the antiviral function of *NbCRVP*, a transient overexpression experiment was carried out using an overexpression vector with 35S promoter. The expression level of *NbCRVP* was detected at 1, 2, 3, and 4 days after transient infiltration of p35S::CRVP-RFP *A. tumefaciens*. The results showed that *NbCRVP* expression level in the 35S::CRVP treatment group innoculated with TMV was 230.39, 69.48, 20.22, and 30.74 times that of the 35S::00 control group also innoculated with TMV, respectively (Fig. 3a). The qRT-PCR results showed that the TMV *CP* mRNA level was decreased by 43%, 28% and 59% at 2, 3, and 4 dpi, respectively, in the 35S::CRVP-TMV group compared with the control group (35S::00-TMV) (Fig. 3b). The WB results further confirmed that TMV CP protein level in the 35S::CRVP-TMV group was lower than that in the control group at 3 dpi (Fig. 3c). Furthermore, the control group exhibited typical symptoms of vein necrosis and wilting, whereas the treatment group showed no such obvious symptoms (Fig. 3d). Inoculation experiments with TMV-30b visually demonstrated a weaker GFP signal in the 35S::CRVP treatment group (Fig. 3e). These results indicated that *NbCRVP* overexpression inhibited TMV infection to *N. benthamiana*.

**Fig. 4:**
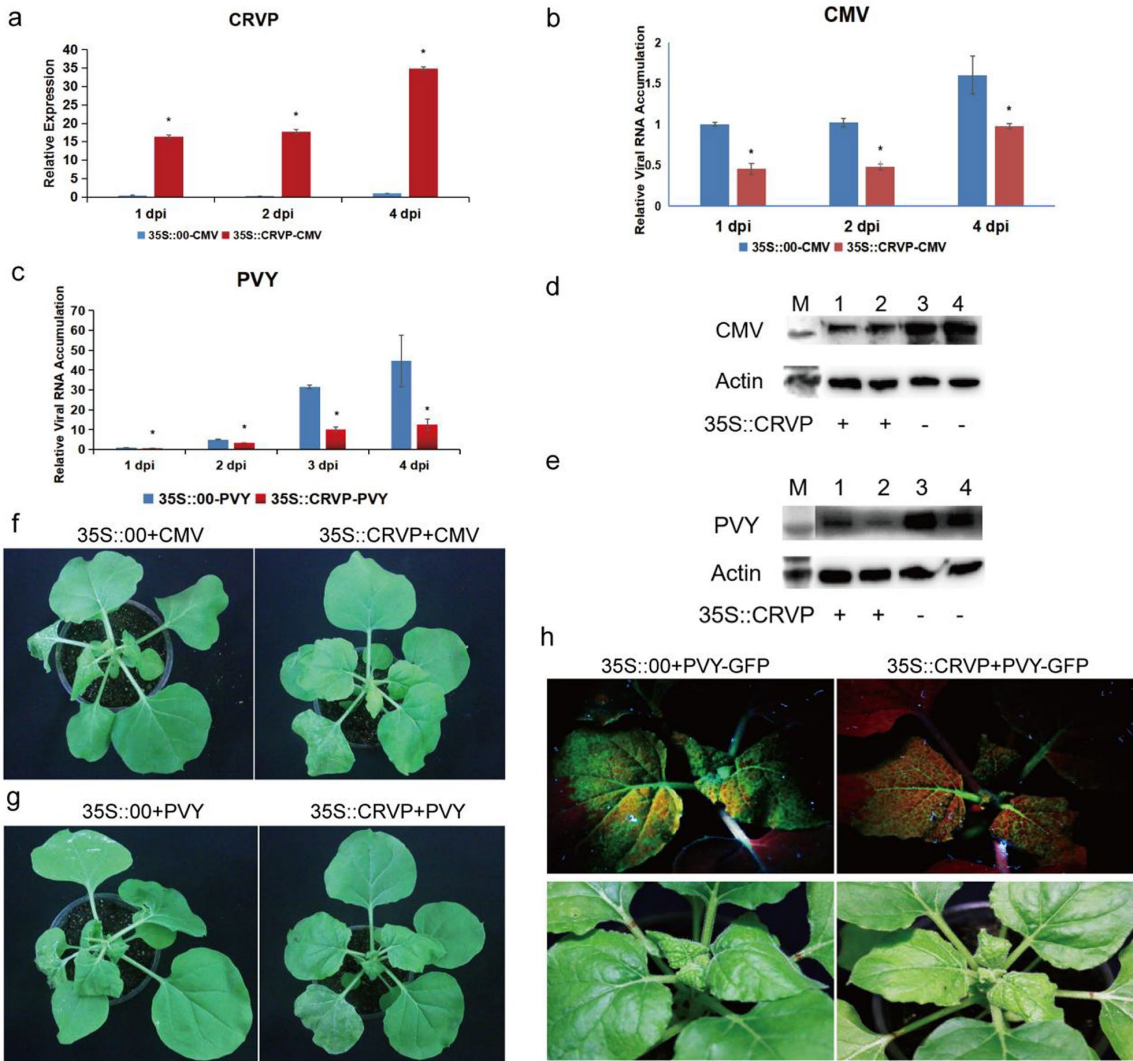
*NbCRVP* overexpression inhibits CMV and PVY infection to *N. benthamiana*. **a** *NbCRVP* levels after its overexpression. **b** CMV CP mRNA levels after *NbCRVP* overexpression at 1, 2, and 4 dpi measured by RT-qPCR. **c** PVY CP mRNA levels after *NbCRVP* overexpression at 1, 2, 3, and 4 dpi, as measured by RT-qPCR. **d** Effect of *NbCRVP* overexpression on CMV infection at the protein level. **e** Effect of *NbCRVP* overexpression on PVY infection at the protein level. **f** Effect of *NbCRVP* overexpression on biological symptoms after CMV infection. **g** Effect of *NbCRVP* overexpression on biological symptoms after PVY infection. **h** Effect of *NbCRVP* overexpression on GFP signals after PVY-GFP infection. The data were analyzed using the independent sample t test, and * indicates significant differences between the two different treatments (*P* < 0.05)

The expression levels of *NbCRVP* in the transient *NbCRVP* overexpression group were 86.11, 71, and 34.88 times that of the control group at 1, 2, and 4 dpi, respectively (Fig. 4a). However, the CMV *CP* levels showed an opposite trend, showing 54%, 53%, and 39% decrease at 1, 2, and 4 dpi, respectively, in the 35S::CRVP group compared with the 35S::00 group, which both inoculated with CMV (Fig. 4b). CMV CP levels were also lower in the treatment group than in the control group at 4 dpi (Fig. 4d). Compared with the 35S::CRVP treatment group inoculated with CMV, the 35S::00 control group inoculated with CMV showed more obvious symptoms of virus infection at 9 dpi (Fig. 4f). These results also indicated that *NbCRVP* overexpression significantly inhibited CMV infection to *N. benthamiana*.

The relative expression levels of *NbCRVP* and PVY *CP* in 35S::CRVP-PVY and 35S::00-PVY groups were also detected after PVY-GFP infection of *N. benthamiana* for 1, 2, 3, and 4 days (Fig. 4c). The PVY CP mRNA levels were decreased by 32%, 68%, and 72% in the 35S::CRVP-PVY group at 2, 3, 4 dpi, respectively, when compared with the 35S::00-PVY group (Fig. 4c). The PVY CP levels were consistent with the results at 4 dpi, shown in Fig. 4e. Compared with the 35S::CRVP treatment group inoculated with PVY, the 35S::00 control group inoculated with PVY showed more obvious symptoms of viral infection at 9 dpi (Fig. 4g), weaker GFP signals were visualized in the 35S::CRVP treatment group than in the control group at 10 dpi, which both infected PVY-GFP (Fig. 4h). These results further illustrated that *NbCRVP* overexpression had a certain inhibitory effect on PVY infection in *N. benthamiana*.

To more comprehensively verify the antiviral function of *NbCRVP*, a multi-infection experiment of TMV, PVY, and CMV was carried out. The expression levels of *NbCRVP* in the 35S::CRVP-virus group were 3022.71, 52.21, 4.99, and 1.83 times that of the 35S::00-virus group at 1, 2, 3, and 4 days after *NbCRVP* overexpression, respectively (Additional file 1: Fig. S4a). The RT-qPCR results showed that TMV *CP* mRNA level was reduced by 87%, 85%, 61%, and 58% in the treatment group compared with the control group at 1, 2, 3, and 4 dpi, respectively (Additional file 1: Fig. S4b). PVY *CP* mRNA level was also reduced by 78%, 96%, and 89% in the treatment group compared with the control group at 1, 2, and 4 dpi, respectively (Additional file 1: Fig. S4b). Similarly, CMV *CP* mRNA level was reduced by 98%, 83%, 92%, and 38% in the treatment group compared with the control group at 1, 2, 3, and 4 dpi, respectively (Additional file 1: Fig. S4b). Moreover, biological symptoms of viral infection were more obvious in the 35S::0-virus group than in the control group (Additional file 1: Fig. S4c). The above results clearly indicated that overexpression of NbCRVP made *Nicotiana benthamiana* less susceptible to RNA virus infection, implying that NbCRVP is a novel protein enhancing the antiviral effects in plants.

### NbCRVP and NbCalB interact to exert enhanced antiviral effects

**Fig. 5:**
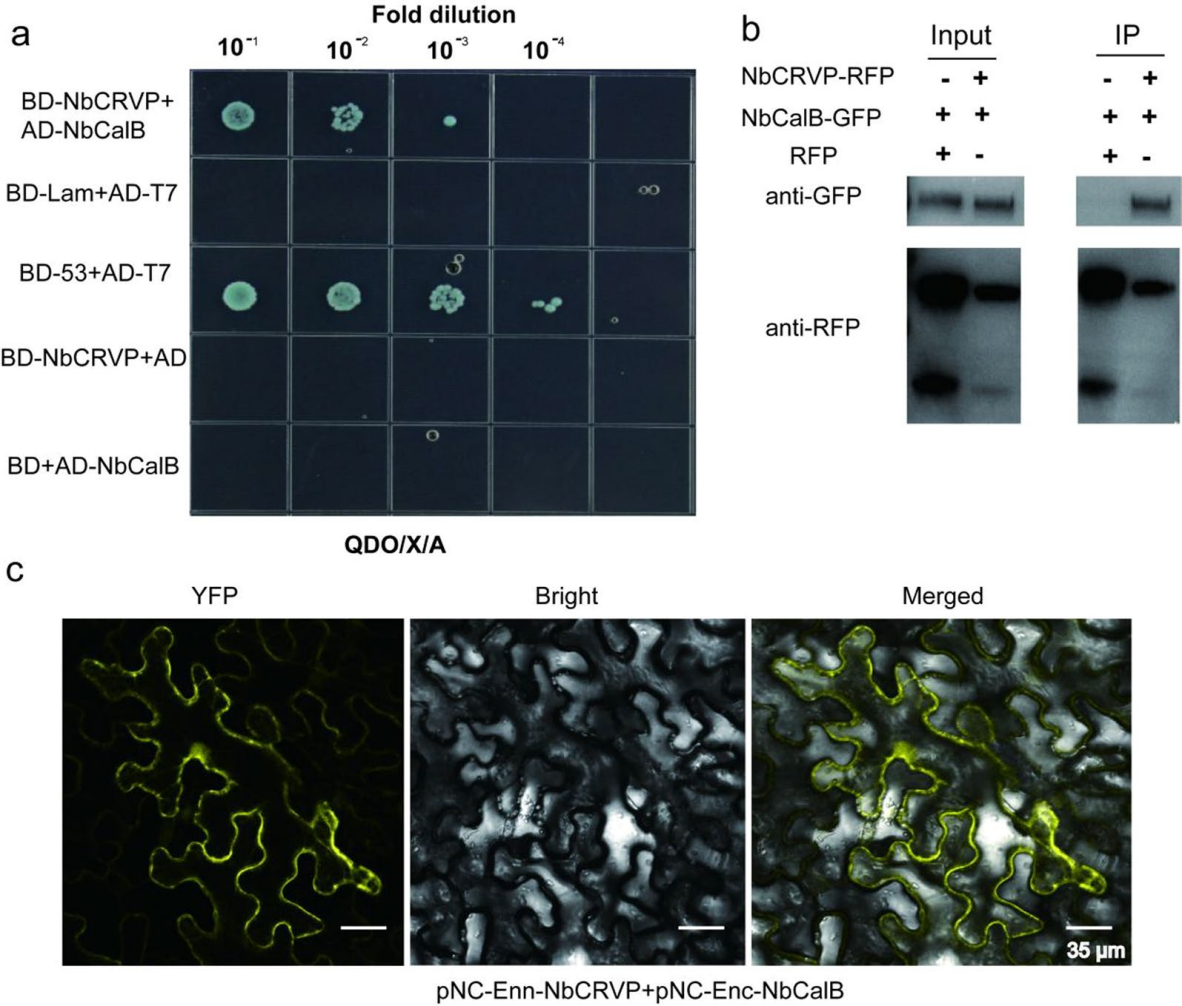
Verification of the interaction between NbCRVP and NbCalB. **a** Y2H point-to-point assay, **b** Co-IP assay, and **c** BIFC assay

To further explore the resistance mechanism of NbCRVP, a Y2H screening test was carried out using NbCRVP as the bait to identify potential interacting proteins. As shown in Additional file 1: Fig. S5, a strong interaction was observed between NbCalB and NbCRVP. The Y2H point-to-point verification results showed that NbCRVP and NbCalB co-transformed strains grew on the SD/-Ade/-His/-Leu/-Trp/X/A (QDO/X/A) plates (Fig. 5a), while both NbCRVP and NbCalB showed no self-activation, indicating an interaction between them (Fig. 5a). The total plant proteins were blotted using an anti-GFP antibody and an anti-RFP antibody, respectively, which imprinted NbCalB-GFP and NbCRVP-RFP bands in both control and treatment groups (Fig. 5b Input), indicating that both NbCalB-GFP and NbCRVP-RFP were expressed. The total plant protein was purified using the RFP magnetic beads, and co-IP results showed that the anti-GFP antibody imprinted the NbCalB-GFP band in the treatment group but not in the control group (Fig. 5b IP). By contrast, the anti-RFP antibody imprinted NbCRVP-RFP bands in both groups (Fig. 5b IP), confirming the interaction between NbCRVP and NbCalB. Moreover, BIFC results showed a strong YFP signal, further demonstrating an interaction between NbCRVP and NbCalB (Fig. 5c).

**Fig. 6:**
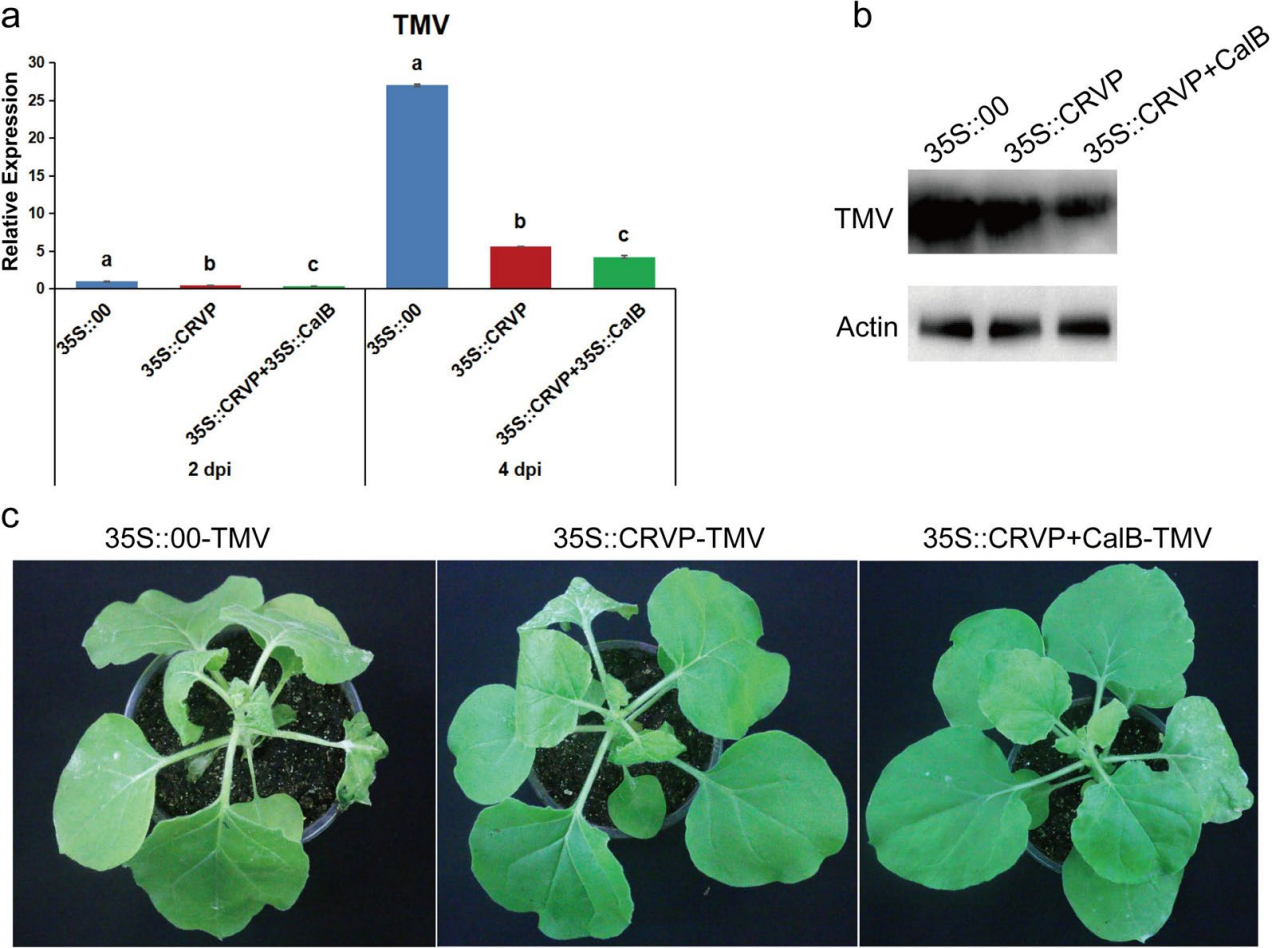
*A. tumefaciens*-mediated co-expression of NbCRVP and NbCalB enhances the antiviral effect. **a** Effects of 35S::CRVP and 35S::CalB co-expression on TMV infection at 2 and 4 dpi, as measured by RT-qPCR, with 35S::00 and 35S::CRVP as the control treatments. The data were analyzed by Duncan’s multiple range tests in the ANOVA program of SPSS. Different letters indicate significant differences among the four treatment groups (*P* < 0.05). **b** Effects of 35S::CRVP and 35S::CalB co-expression on TMV infection at 4 dpi, as measured using WB. **c** Effects of 35S::CRVP and 35S::CalB co-expression on biological symptoms between the three different treatments

To ascertain the impact of the interaction between NbCRVP and NbCalB on viral infection, *A. tumefaciens* were transformed with expression vectors 35S::00, 35S::CRVP, or 35S::CRVP + 35S::CalB and infected with TMV. RT-qPCR results showed that TMV *CP* mRNA levels were down-regulated in 35S::CRVP and 35S::CRVP + 35S::CalB transformed *A. tumefaciens* by 53% and 65% at 2 dpi, and 79% and 84% at 4 dpi, respectively, compared with that of 35S::00-transformed control *A. tumefaciens* (Fig. 6a). Western blotting results for TMV CP protein levels were consistent with the changes in mRNA levels at 4 dpi (Fig. 6b). The control group 35S::00 inoculated with TMV showed obvious symptoms of vein necrosis and wilting, and the top new leaves of the 35S::CRVP treated group showed curling symptoms. However, the viral infection symptoms of 35S::CRVP + CalB group were the least noticeable (Fig. 6c). These results indicated that NbCRVP and NbCalB interact to exert enhanced antiviral effects.

### Preparation and characterization of PAMAM@pDNA nanocomposites

**Fig. 7:**
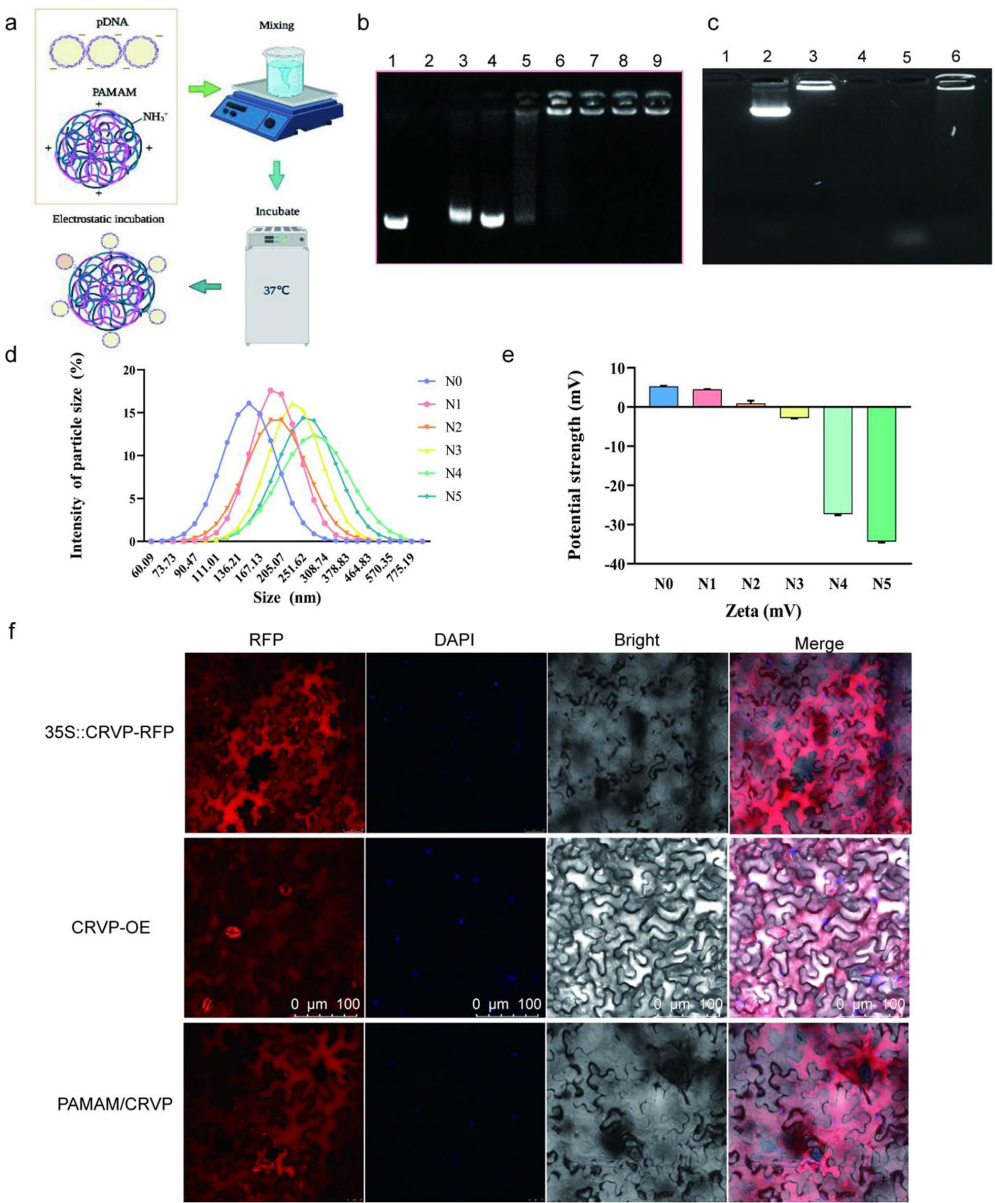
Preparation and characterization of PAMAM@CRVP nanocomposites. **a** The process of preparing PAMAM@CRVP nanocomposites. **b** The maximum plasmid loading capacity of PAMAM. Lane 1: mock 35S::CRVP plasmids; Lane 2: mock PAMAM; and Lanes 3–9: PAMAM@CRVP nanocomposites with N/P ratios of 1:5, 1:3, 1:2, 1:1, 2:1, 3:1, and 5:1. **c** DNase treatment to detect the stability of PAMAM@DNA. Lane 1: PAMAM; Lane 2: plasmids; Lane 3: PAMAM@DNA; Lane 4: PAMAM + DNase; Lane 5: plasmids + DNase; Lane 6: PAMAM@DNA+DNase. **d** The particle size of PAMAM@CRVP nanocomposites with different N/P ratios. N0: mock PAMAM; N1-5: nanocomposites with N/P ratios of 1:3, 1:2, 1:1, 2:1, and 3:1, respectively. **e** The zeta potentials of PAMAM@CRVP nanocomposites with different N/P ratios. N0: mock PAMAM; N1-5: nanocomposites with N/P ratios of 1:3, 1:2, 1:1, 2:1, and 3:1, respectively. **f** Laser confocal observation of NbCRVP expression in 35S::CRVP-RFP *A. tumefaciens*-infiltrated, CRVP-OE and PAMAM@CRVP nanocomposites treated plants.

To achieve efficient delivery and expression of the resistant protein, we selected PAMAM to prepare plasmids-coated nanocomposites. The optimal enveloping condition was achieved by incubating at 37 ℃ for 30 min. PAMAM and expression plasmids can be combined together to form nanocomposites under the action of electrostatic binding force (Fig. 7a). Agarose gel electrophoresis was used to detect the binding capacity of PAMAM to plasmids under different N/P ratios. When the N/P ratios were 1:5 and 1:3, gel electrophoresis showed representative bands with mobility similar to the Mock-35S::CRVP plasmid. When the N/P ratio was 1:2, the 35S::CRVP plasmid could be completely coated by PAMAM nanocarriers. Overall, the results indicated that at the N/P ratio of 1:2, PAMAM exhibited the maximum loading limit (Fig. 7b). The TEM and SEM images showed that PAMAM@CRVP complexes were regularly dispersed and round and had the core size from 25 to 100 nm (Additional file 1: Fig. S6a, b). The results of DNase treatment showed that the plasmids were completely degraded after DNase treatment (37 °C, 10 min), while the PAMAM@pDNA nanocomposites were only partially degraded after DNase treatment compared with the control group, indicating that PAMAM could protect DNA from degradation by DNase (Fig. 7c).

The diameter of the nanocomposites was further tested using dynamic light scattering (DLS). PAMAM@CRVP nanocomposites at N/P ratios of 1:3, 1:2, 1:1, 2:1, and 3:1 as well as mock PAMAM had an average size of 196.8 nm, 227.7 nm, 201.8 nm, 263.7 nm, 267.4 nm and 135.6 nm (Fig. 7d) and particle dispersion indices of 0.184, 0.442, 0.140, 0.195, 0.436 and 0.608, respectively (Additional file 2: Table S2). The zeta potential value of the PAMAM@CRVP nanocomposites was also examined in more detail using a potentiometric analyzer. The average zeta potentials of mock PAMAM and PAMAM@CRVP nanocomposites at N/P ratios of 1:3, 1:2, 1:1, 2:1, and 3:1 were 5.927 mV, 4.857 mV, 0.926 mV, 2.835 mV, -26.067 mV, and -33.667 mV, respectively (Fig. 7e), showing a decrease trend upon incubation with negatively charged DNAs, and confirming that DNAs were effectively combinated with PAMAM. Laser confocal microscopy was used to more intuitively detect the expression of NbCRVP in plants treated with 35S::CRVP-RFP *A. tumefaciens*, CRVP-OE, and PAMAM@CRVP nanocomposites. The red fluorescence signal of NbCRVP-RFP fusion proteins was observed in all three treatment groups, indicating that the PAMAM@CRVP nanocomplexs have been successfully delivered into the plant cells (Fig. 7f). Protoplasts prepared after infiltration of PAMAM@CRVP:CalB nanocomposites in *Solanum lycopersicum* and *Capsicum annuum* also confirmed the expression of NbCRVP and NbCalB (Additional file 1: Fig S7). Continuous monitoring found that PAMAM-mediated gene expression was transient. On the 9th day after the PAMAM@CRVP:CalB nanocomplex infiltrated the plant leaves, the CRVP-RFP and CalB-GFP fluorescence signals were no longer detectable in the cells (Additional file 1: Fig S8a). However, the in vitro shelf life of these PAMAM@pDNA nanocomplexes could reach 28 days (Additional file 1: Fig S8b).

### Optimization of the PAMAM@NbCRVP:NbCalB ratio for the best antiviral effects

**Fig. 8:**
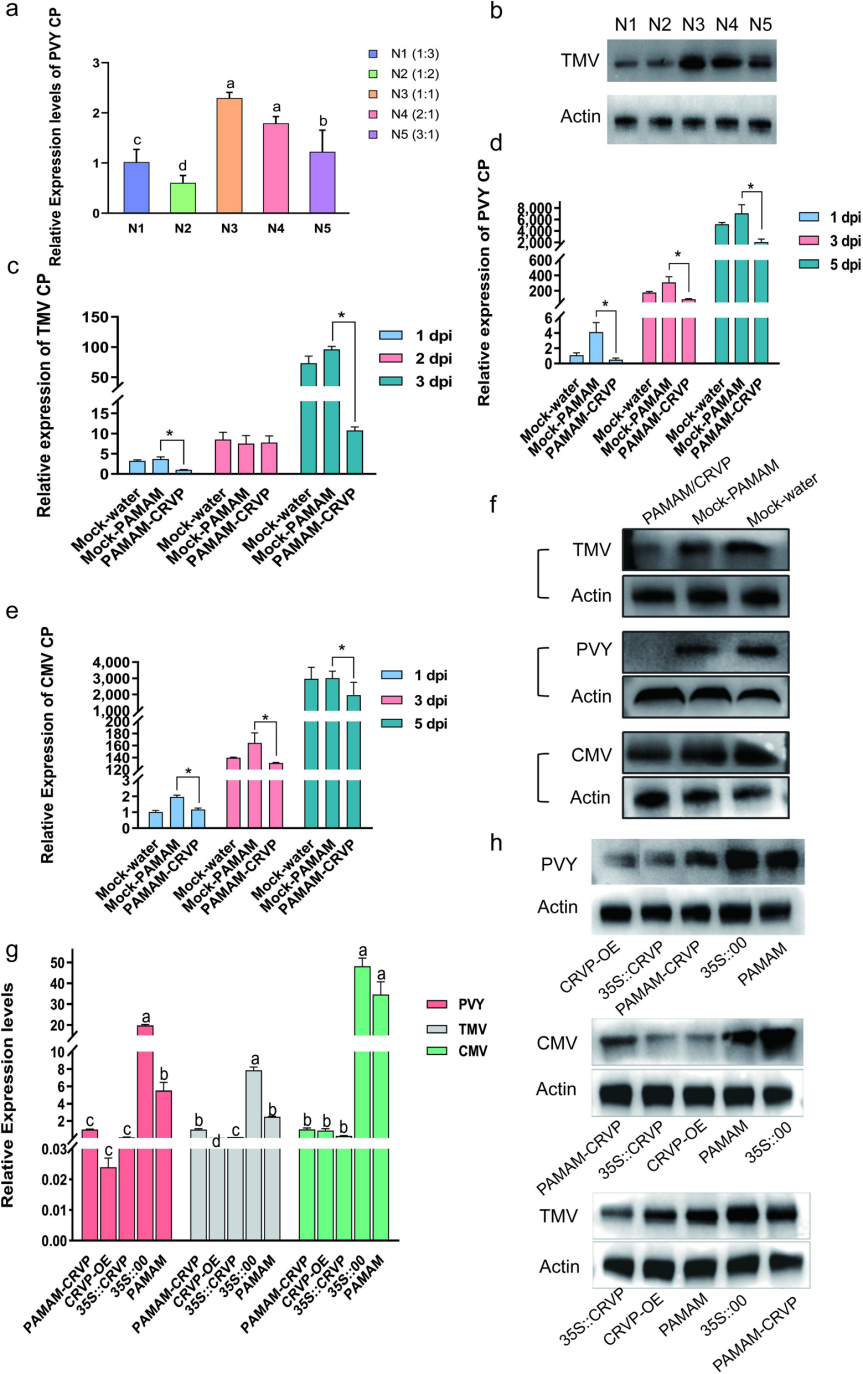
Antiviral effects of PAMAM@CRVP nanocomposites. **a** The inhibitory effects of PAMAM@CRVP nanocomposites with different N/P ratios on PVY infection, as detected using RT-qPCR. **b** The inhibitory effects of PAMAM@CRVP nanocomposites with different N/P ratios on PVY infection, as measured by WB. **c** The inhibitory effect of PAMAM@CRVP nanocomposites on TMV infection at 1, 2, and 3 dpi, as detected by RT-qPCR. **d** The inhibitory effect of PAMAM@CRVP nanocomposites on PVY infection at 1, 3, and 5 dpi, as detected by RT-qPCR. **e** The inhibitory effect of PAMAM@CRVP nanocomposites on CMV infection at 1, 3, and 5 dpi, as detected by RT-qPCR. **f** The inhibitory effect of PAMAM@CRVP nanocomposites on PVY CMV and TMV infections, as detected by WB. **g** The inhibitory effect of PAMAM@CRVP nanocomposites on PVY CMV and TMV infections comparing with CRVP-OE, 35S::CRVP, and 35S::00, PAMAM, as detected by RT-qPCR. **h** The inhibitory effect of PAMAM@CRVP nanocomposites on PVY CMV and TMV infections, comparing with CRVP-OE, 35S::CRVP, 35S::00, and PAMAM, as measured by WB. Data shown in **a** and **g** were analyzed using Duncan’s multiple range tests in the ANOVA program of SPSS, with different letters indicating significant differences among different treatment groups (*P* < 0.05). Data shown in **c**, **d** and **e** were analyzed using the independent sample t test, with * indicating significant differences between two treatment groups (*P* < 0.05).

To determine the optimal ratio of PAMAM nanocarriers to CRVP plasmids, we tested their effects on PVY infection using RT-qPCR at the N/P ratios of 1:3, 1:2, 1:1, 2:1, and 3:1. It was found that PAMAM@CRVP had the best inhibitory effect at the N/P ratio of 1:2 (Fig. 8a), which was further verified using WB (Fig. 8b). Therefore, the N/P ratio of 1:2 was selected as the optimal ratio for subsequent preparation of nanocomposites.

To verify the anti-disease effect of PAMAM@CRVP nanocomposites, the PAMAM@CRVP nanocomposites were tested against TMV, PVY, and CMV infection. RT-qPCR data showed that (1) TMV *CP* mRNA level was reduced by 73% and 89% at 1 and 3 dpi, respectively, in the PAMAM@CRVP group compared with the control mock-PAMAM group (Fig. 8c); (2) PVY *CP* mRNA level was reduced by 88%, 72%, and 71% at 1, 3, and 5 dpi, respectively, compared with the control mock-PAMAM group (Fig. 8d); and (3) CMV *CP* mRNA level was reduced by 40%, 20%, and 35% at 1, 3, and 5 dpi, respectively, compared with the control mock-PAMAM group (Fig. 8e). WB results also revealed decreased levels of TMV CP at 3 dpi, PVY CP at 3 dpi, and CMV CP at 5 dpi, consistent with the results at mRNA levels (Fig. 8f). These results indicated that PAMAM@CRVP nanocomposites successfully delivered the disease-resistant protein DNA and exerted virus-resistant effect. Furthermore, *A. tumefaciens*-mediated treatment of plants with 35S::CRVP, and PAMAM@CRVP nanocomposites inhibited TMV, PVY, and CMV infection, comparable to the inhibitory effect with transgenic plants (Fig. 8g, h). These data further confirmed that our study provided a new delivery platform for antiviral proteins.

**Fig. 9:**
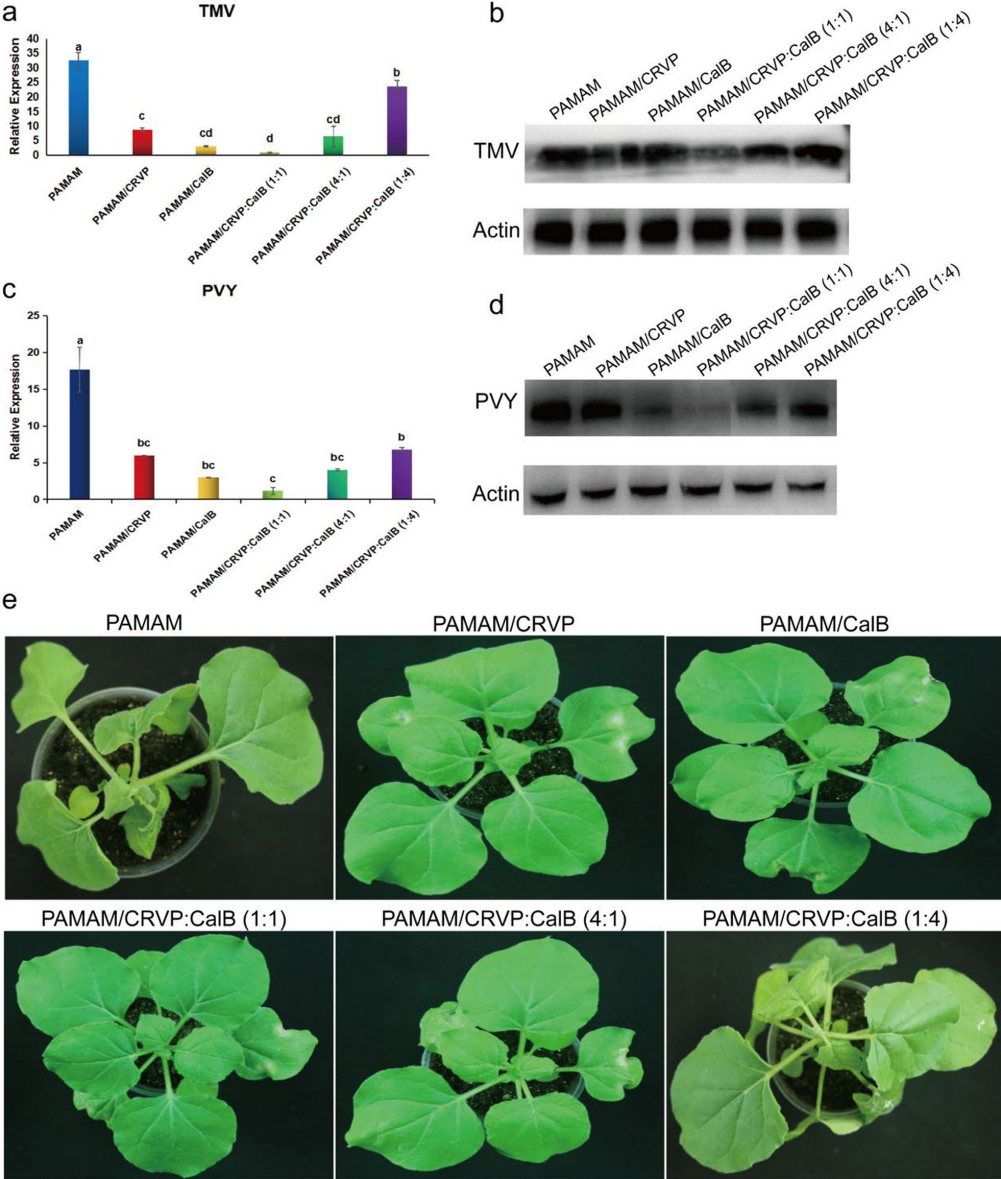
Interaction between NbCRVP and NbCalB has a synergistic anti-disease effect. **a** Effect of PAMAM nanocomposites with different NbCRVP:NbCalB ratios on TMV infection at 4 dpi, as detected by RT-qPCR. **b** Effects of PAMAM nanocomposites with different NbCRVP:NbCalB ratios on TMV infection at 4 dpi, as detected by WB. **c** Effect of PAMAM nanocomposites with different NbCRVP:NbCalB ratios on PVY infection at 4 dpi, as detected by RT-qPCR. **d** Effects of PAMAM nanocomposites with different NbCRVP:NbCalB ratios on PVY infection at 4 dpi, as detected by WB. **e** Effect of PAMAM nanocomposites with different NbCRVP:NbCalB ratios on the symptoms of TMV infection at 4 dpi. The data shown in **a** and **c** were analyzed by Duncan’s multiple range tests in the ANOVA program of SPSS, with different letters indicating significant differences among different treatment groups (*P* < 0.05).

To clarify the optimal ratio of NbCRVP and NbCalB and their synergistic anti-disease effect, we set up several treatments, including mock-PAMAM, PAMAM@CRVP, PAMAM/CalB, PAMAM@CRVP:CalB (1:1), PAMAM@CRVP:CalB (4:1) and PAMAM@CRVP:CalB (1:4). qRT-PCR results showed that PAMAM@CRVP, PAMAM/CalB, PAMAM@CRVP:CalB (1:1), PAMAM@CRVP:CalB (4:1), and PAMAM@CRVP:CalB (1:4) treatments downregulated the expression of TMV *CP* mRNA at 4 dpi by 73%, 90%, 97%, 80%, and 27%, respectively, with PAMAM@CRVP:CalB (1:1) showing the best resistance effect (Fig. 9a). WB results further verified that TMV CP protein content was the lowest in the PAMAM@CRVP:CalB (1:1) group (Fig. 9b). Moreover, The PAMAM control group showed obvious symptoms of wilting and necrosis, while the PAMAM@CRVP:CalB (1:1) treatment group displayed no obvious virus infection symptoms (Fig. 9e). We also tested the effects of the above six treatment groups on PVY infection. PVY *CP* mRNA (Fig. 9c) and protein (Fig. 9d) levels were the lowest in the PAMAM@CRVP:CalB (1:1) group, indicating the interaction between NbCRVP and NbCalB had a synergistic anti-disease effect, and NbCRVP:NbCalB at the ratio of 1:1 had the best anti-disease effect.

## Discussion

Plant viral diseases seriously threaten commercial crops worldwide, leading to decreased quality and yield [32, 33]. Breeding disease-resistant varieties is currently the most effective modality to control these diseases, with the key issues being mining and efficiently utilizing disease-resistant genes. In this study, we, for the first time, identified a novel plant cysteine-rich venom protein (NbCRVP), clarified its role in inhibiting RNA viral infections, and revealed its interaction protein NbCalB. PAMAM was selected for producing nanocarriers that could deliver plasmids expressing NbCRVP and NbCalB, leading to its antiviral effects. Subsequent verification experiments showed that the optimal ratio of NbCRVP::NbCalB (1:1) in PAMAM@CRVP::CalB resulted in enhanced antiviral effects.

CRISP proteins are typically named after the organisms they were isolated from. The first CRISP described in reptiles was isolated from the skin secretion of the lizard *Heloderma horridum* and named helodermin [34]. Other examples include the protein patagonin isolated from the venom of the snake *Philodryas patagoniensis* [35], latisemin isolated from the sea snake *Laticauda semifasciata*, tigrin isolated from *Rhabdophis tigrinus tigrinus* [36] and ablomin isolated from *Gloydius blomhoff* [36]. In this study, NbCRVP was identified for the first time from the model plant *N. benthamiana*. A global analysis of *NbCRVP* expression pattern showed that *NbCRVP* was upregulated under viral infection and present in the root, stem, leaf, and flower tissues of *N. benthamiana*, with the highest content in flower. Moreover, NbCRVP was distributed in the entire cell outline, but not in the nucleus. The present study is the first report on the characterization of a novel CRISP identified from plants, denoted as NbCRVP.

CRISPs belong to a large class of proteins ubiquitous in vertebrates that participate in various biological processes [37–40]. They have been reported in pancreatic tissues, salivary glands, and reproductive tracts [11, 13, 41], and reptile venom ducts [8–10]. These proteins have multiple biological activities, including inhibiting various ion channels and inducing proteolysis and prey paralysis. Snake venom has been used to kill HIV [42], *Plasmodium falciparum* [43] and *Leishmania* [44]. Crude venom from the snake *Bothrops* inhibits the growth of *Leishmania major* promastigotes and *T*. *cruzi* epimastigotes [45] and induces programmed cell death in *T*. *cruzi* [46]. The antiprotozoal activity of CRISP has been reported from Crovirin, which has shown good activity against Trypanosoma and Leishmania [47]. However, the biological activity of most CRISPs has not been confirmed. In this study, the VIGS test initially suggested that NbCRVP can inhibit TMV and PVY infections. The overexpression test displayed that viral CP mRNA and protein levels were lower in the *NbCRVP* overexpression treatment group than in the control group under infection of TMV, PVY, and CMV, respectively, further verifying that NbCRVP can inhibit the infection of RNA viruses in plants. This is the first report on the antiviral function of NbCRVP in plants, making *NbCRVP* a novel antiviral gene in plants.

Currently, the most urgent issue to solve is the efficient delivery and expression of resistant genes in plant disease resistance. *Agrobacterium*-mediated delivery is commonly used for this purpose [48] but has limitations. It can only deliver resistant genes to a narrow range of plant species and tissue types, does not perform DNA-free and transgene editing [49], and is not suitable for high-throughput applications. Despite decades of advances in biotechnology, most plant species remain difficult to transform genetically [50]. One bottleneck is the delivery of biomolecules into plant cells, hindered by rigid and multilayered cell walls. There are few delivery tools available to transfer biomolecules into plant cells, each with significant limitations. Biolistic particle delivery, also known as gene gun [51], is another commonly used tool that can deliver biomolecules to a wider range of plant species but has its own limitations, such as damage to plant tissues with high bombardment pressure, limited specimen size, positioning in the biolistic chamber, and the requirement to use large amounts of DNA for efficient delivery [51]. In recent years, efficient diffusion of nanomaterial-based delivery of biomolecules into tissues and organs of intact plants of multiple species, which overcomes the drawbacks of the two techniques above. Efficient DNA delivery and strong protein expression were achieved without transgene integration in *N. benthamiana*, arugula, wheat, and Gossypium hirsutum (cotton) leaf and arugula protoplasts [52]. And small interfering RNA (siRNA) were delivered to *N. benthamiana* leaves and silenced genes with 95% efficiency [52]. Here, NbCalB, an interacting protein of NbCRVP, was identified. *A. tumefaciens*-mediated transient overexpression experiments showed that co-expression of NbCRVP and NbCalB endowed *N. benthamiana* with enhanced antiviral effect to TMV infection. The PAMAM@CRVP:CalB nanocomposites were further prepared, the average particle size of the nanocomposites was 227.0 nm, and the average zeta potentials of PAMAM@CRVP:CalB nanocomposites was 0.926 mV (Additional file 1: Fig S9). The prepared nanocomplexes has low cytotoxicity (Additional file 1: Fig S10), and could protect the plasmids from nuclease degradation (Fig. 7c). The successful delivery and protein expression of PAMAM@CRVP:CalB nanocomplex was confirmed not only in *N. benthamiana* but also in tomato and pepper (Fig. 7f; Additional file 1: S7, 8). Further verification of the virus resistance showed that the CRVP::CalB binary DNA delivered by PAMAM had the best antiviral effect at a ratio of 1:1. It may be that the interaction between the two proteins is the strongest at this ratio, and the synergistic anti-disease function is fully exerted. Reference has reported a schematic diagram of the intracellular transport and expression of genes carried by carbon nanotubes (CNTs): unloading in the cell or unloading in the nucleus requires the initiation of transcription in the nucleus [52]. Plant cells transfected with the gene expression cassette/CPP complex also showed efficient translocation of DNA molecules into the nucleus and transient expression of foreign genes [53–55]. Regarding the mechanism of PAMAM-mediated gene delivery into cells, PAMAM@pDNA nanocomposites electrostatically attaches to negatively charged phospholipids on the outer surface of the cell membrane, stimulating the complex’s uptake through energy-dependent endocytosis. The hidden amino groups (inside the dendrimers) act as a “proton sponge”, helping DNA to be released from endosomes into the cytoplasm and translocated to the nucleus, where it is transcribed into mRNA. The mRNA template is transported into the cytosol, where it is translated into a functional protein [29, 56, 57]. However, no genomic integration was occurred (Additional file 1: Fig S11). Nonetheless, more detailed delivery and release mechanisms require further study.

## Conclusions

This is the first ever identification and functional analysis of NbCRVP and its interacted protein NbCalB. Additionally, herein, for the first time, achieved non-transgenic, nanocarrier-mediated efficient delivery of DNA expressing two interactive antiviral proteins, laying the foundation on new theories and control strategies for plant disease controls.

## Materials and methods

### Plant materials and viral strains

Two sets of *N. benthamiana* plants were used in this study: (a) wild-type (seeds preserved in our laboratory); (b) *NbCRVP*-overexpressing (OE) transgenic plants with a red fluorescent protein (RFP) tag constructed by Wuhan Edgene Biotechnology Co., Ltd. (Wuhan, China). All these materials were grown at 25 °C in chambers with 50–60% humidity and a 16-h/8-h light/dark photoperiod. The following strains: TMV-U1, TMV-30b, PVY-GFP [30], PVY^N:O^, and CMV IB were used for inoculation.

### Cloning the open-reading frame sequence of *NbCRVP* and vector construction

Total plant RNAs were extracted from 100 mg of fresh samples using TRIzol (Vazyme, Nanjing, China) and reverse transcribed to cDNA using reverse transcription reagents following the manufacturer’s instructions (Vazyme, Nanjing, China). The open-reading frame sequence of *NbCRVP* was then amplified using primers CRVP-F/CRVP-R.

To construct the p35S::CRVP-RFP expression vector, the coding sequence (CDS) of *NbCRVP* was first subcloned into the intermediate vector pFu46-RFP via homologous recombination and then cloned into pEarleygate via LR reaction as described previously [30].

The pTRV2::CRVP vector was constructed by amplifying a 200–300-bp fragment from the *NbCRVP* CDS sequence and inserting it into the pTRV2 vector through in-fusion technology [30].

To construct the p35S::CalB-GFP expression vector, the *NbCalB* CDS was first subcloned into the intermediate vector pFu28-GFP via homologous recombination and then cloned into pEarleygate via the LR reaction as described previously [30].

The pBD-NbCRVP vector was constructed by inserting the *NbCRVP* CDS into the pBKT7 vector.

The pAD-NbCalB vector was constructed by inserting the *NbCalB* CDS into the pADT7 vector.

The pEnn-NbCRVP vector was constructed by inserting the *NbCRVP* CDS into the pEnn vector.

The pEnc-NbCalB vector was constructed by inserting the *NbCalB* CDS into the pEnc vector.

### Virus-induced gene silencing (VIGS)

Four-week-old *N. benthamiana* plants were co-infiltrated with *A. tumefaciens* carrying pTRV1 and pTRV2::CRVP at a ratio of 1:1. In addition, plants at the same age co-infiltrated with *A. tumefaciens* carrying pTRV1 and pTRV2::00 or pTRV1 and pTRV2::PDS were used as the negative and positive controls, respectively [31]. Silencing efficiency was measured 14 days after the treatment. To investigate the effects of gene silencing on viral infection, the viruses were inoculated on the 15th day, and samples were collected at various time points to detect the relative virus expression.

### *Agrobacterium*-mediated overexpression assay

The constructed p35S::CRVP::RFP and p35S::00::RFP expression plasmids were transformed into *A. tumefaciens* LBA4404, respectively. The transformed *A. tumefaciens* was cultured in buffer with 10 mmol/L MES, 200 μmol/L AS and 10 mmol/L MgCl_2_, until the OD600 reached 0.8. To verify the effect of *NbCRVP* overexpression on virus infection, 5-week-old *N. benthamiana* plants with the same growth pattern were first prepared, which were then infiltrated with 35S::CRVP (the treatment group) and 35S::00 (the control group) *A. tumefaciens* using a needle-free syringe, respectively. After 4 h, the virus was inoculated to the treated leaves through friction, and samples were collected to detect the differences in viral content between the treatment group and the control group.

### Yeast two-hybrid (Y2H) verification

To perform the point-to-point verification assay, Y2H yeast competent cells were co-transformed with pBD-NbCRVP and pAD-NbCalB plasmids and cultured on an SD/-Leu/-Trp/x-a-gal/Aba (DDO/X/A) medium for 3–5 days. Single colonies were picked, inoculated to the QDO/X/A (200 ng/mL) medium, and cultured for 3–5 days. Corresponding single colonies growing on the QDO/X/A (200 ng/mL) medium were subjected to gradient dilution and further cultured for 3–5 days. Interaction between the foreign and target proteins was verified based on the growth of each transformation combination on the plate.

### Co-immunoprecipitation (Co-IP) assay

The constructed p35S::CRVP::RFP and p35S::CalB::GFP expression plasmids were transformed into *A. tumefaciens* LBA4404, respectively. The transformed *A. tumefaciens* was cultured in buffer with 10 mmol/L MES, 200 μmol/L AS and 10 mmol/L MgCl_2_. When an OD600 of 0.8 was achieved, *A. tumefaciens* were used to co-infiltrate the lower surface of 5-week-old *N. benthamiana* leaves using a needle-free syringe. In addition, *N. benthamiana* leaves co-infiltrated with *A. tumefaciens* transformed with p35S::00::RFP and p35S:CalB::GFP expression vectors were used as the controls. At 3–4 days after infiltration, *N. benthamiana* leaves were collected to extract total plant proteins. The proteins were purified using magnetic beads coupled with an anti-RFP antibody (ABclonal, Wuhan, China) and subjected to SDS-PAGE before immunoblotting with an anti-GFP antibody (ABclonal, Wuhan, China) to verify the interaction between NbCRVP and NbCalB based on the size difference between the treatment and control protein bands.

### Bimolecular fluorescence complementation (BIFC) assay

The constructed pEnc-NbCRVP and pEnn-NbCalB plasmids were transferred into *A. tumefaciens* GV3101, respectively. The transformed *A. tumefaciens* cultures were mixed at 1:1 ratio and used to infiltrate *N. benthamiana*. After 48 h, the presence or absence of YFP signals was observed under a laser confocal microscope.

### PAMAM@pDNA complex preparation

PAMAM dendrimer, ethylenediamine core carrying 32 amino groups, 3.0 solution (product number P860780) was used as the pDNA carrier. The PAMAM@pDNA complex was assembled as spherical nanoparticles via the electrostatic force between the positive charges on the amino groups of PAMAM and the negative charges of the plasmid nucleotides. In brief, 200 ng/μL PAMAM solution was first prepared by uniformly adding 1.1587 μL PAMAM solvent dropwise into 1000 mL sterile water under stirring with a magnetic stirrer for 6 h. The plasmid was diluted to a uniform concentration of 200 ng/uL, and then different proportions of PAMAM@pDNA nanocomplexes were prepared according to different volume ratios. The PAMAM@pDNA complex was then incubated at 37 °C for 30 min to promote the formation of nanoparticles. The resulting nanoparticle/plasmid complexes were used for subsequent experiments.

### Characterization of PAMAM@pDNA nanocomposites

To evaluate PAMAM’s ability to bind plasmids, PAMAM@CRVP nanocomposites were prepared by keeping the CRVP plasmid concentration at 200 ng/μL with varying molar ratios of PAMAM to plasmids, or the N/P ratios. The nanocomposite samples were subjected to agarose gel electrophoresis at 80 V for 30 min, and the binding efficiency of PAMAM was determined based on the migration of DNA bands. To further analyze the morphological characteristics, PAMAM@CRVP nanocomposites were placed on a copper film with a carbon mesh and observed with a scanning electron microscope (SEM) with vacuum and a transmission electron microscope (TEM) after drying. The 2D projections of the nanocomposites were scanned and used to project their surface 3D images. The potential or the ζ-electromotive force and particle size of the PAMAM@CRVP nanocomposites at different N/P ratios were determined using a potential particle size measurement analyzer (Zetasizer Nan ZS90, UK). DNase I(TransGen Biotech, Beijing, China) treatment at 37 °C for 10 min to detect the stability of the nanocomposites.

### Real-time fluorescence relative quantitative PCR (RT-qPCR)

Total RNAs were extracted and used as the template to synthesize cDNA samples following the program of the reverse transcription kit (Vazyme, Nanjing, China). The relative mRNA expression levels of target genes were quantitatively detected following the instructions of the real-time fluorescence quantitative PCR kit (Vazyme, Nanjing, China). The primer sets TMV-F/TMV-R, PVY-F/PVY-R, CMV-F/CMV-R, and β-ActinQF/β-ActinQR (Additional file 2: Table S1) were used to amplify TMV *CP*, PVY *CP*, CMV *CP*, and *β-actin,* respectively. The 2^−ΔΔCt^ method was employed to calculate the relative expression levels of these genes. All experiments were repeated three times with three biological replicates. Data are expressed as mean ± standard deviation of at least three independent experiments. Statistical analyses were performed using SPSS (v21, IBM, Armonk, NY, USA) with Duncan’s multiple range test analysis of variance (ANOVA) and independent sample *t*-test. Statistical significance was set at *p* < 0.05.

### Western blotting (WB)

To detect the presence of viral proteins, total plant proteins were extracted using protein extraction kit (ABclonal, Wuhan, China) and subjected to WB using virus-specific antibodies against TMV, PVY, and CMV (Agdia, Elkhart, IN, USA), respectively. In addition, β-actin was used as the internal reference and detected using β-actin antibodies (ABclonal, Wuhan, China).

### Supplementary Information

**Additional file 1**: **Figure S1. **The expression pattern of *NbCRVP*. (a) Expression of *NbCRVP* under the stress of viral infection at 1, 3, 5, 7 dpi. The data were analyzed by Duncan’s multiple range tests in the ANOVA program of SPSS, different letters indicate that values of the four treatments were significantly different (*P* < 0.05), which were the same as b, c, d. (b) The *NbCRVP* expression trends in flower, stem, leaf and root. (c) The *NbCRVP* expression trends after spraying with 0.5 mM SA, 0.05 mM ethephon, or 0.1 mM Me-JA, water with 0.02% Tween 20 was used as a control. (d) The expression of *NbCRVP* after silencing the key signaling genes, *NPR1*, *COI1* and *EIN2*. (e) Subcellular distribution of NbCRVP observed in healthy *N. benthamiana* and virus infected *N. benthamiana.* **Figure S2**. Subcellular distribution of NbCRVP using DAPI as a nuclear localization marker. **Figure S3**. The silencing efficiency of *NbCRVP*. (a) Phenotype after 14 days of silencing *NbCRVP*. (b) The silencing efficiency was detected after 14 days of treatment. **Figure S4** Overexpression *NbCRVP* on multi-infection of TMV, PVY and CMV. (a) The detection of *NbCRVP* after overexpression *NbCRVP.* The data were analyzed with independent sample t test, * indicated that values of the two treatments were significantly different (*P* < 0.05), the same as b. (b) Effect of overexpression *NbCRVP* on multi-infection of TMV, PVY and CMV detected by RT-qPCR at 1, 2, 3, 4 dpi. (c) Differences in biological symptoms of overexpression *NbCRVP* on multi-infection of TMV, PVY and CMV. **Figure S5** Screening of interactive proteins for NbCRVP in yeast two-hybrid assay. (a) Screening of interacting proteins by dot culture on QDO/X/A. (b) PCR amplification, gel electrophoresis, and sequencing to identify interacting proteins. **Figure S6** SEM and TEM images of PAMAM@CRVP nanocomposites. **Figure S7** Detect the expression of PAMAM@CRVP:CalB in the *Solanum lycopersicum* and *Capsicum annuum* protoplast. (a) Detect the expression of PAMAM@CRVP:CalB in *Solanum lycopersicum.* (b) Detect the expression of PAMAM@CRVP:CalB in *Capsicum annuum.* **Figure S8** In vitro shelf life and delivery expression of PAMAM@pDNA nanocomplexes in plant cells. (a) Monitoring the delivery and expression of PAMAM@pDNA nanocomplex in plant cells under laser confocal. (b) Monitor the stability of PAMAM@pDNA nanocomplexes in vitro. **Figure S9** Characterization of PAMAM@CRVP:CalB nanocomposites. (a) Average particle size distribution of PAMAM@CRVP:CalB nanocomposites. (b) Intensity distribution of Zeta potential of PAMAM@CRVP:CalB nanocomposites. **Figure S10** Toxicity assay of PAMAM@CRVP:CalB nanocomposites. (a) Dry cell weight determination treated with PAMAM@CRVP:CalB nanocomposites. (b) cell mortality rate determination treated with PAMAM@CRVP:CalB nanocomposites. **Figure S11** Validation that PAMAM-mediated resistance protein delivery was non-transgenic. Lane 1 was PAMAM@CRVP:CalB-treated wild-type *N. benthamiana* seeds, Lane 2 was PAMAM@CRVP:CalB-treated NbCRVP-OE seeds.

**Additional file 2**: **Table S1.** Primers used in this study. **Table S2.** Characterization data of PAMAM@CRVP nanocomposites.

## Data Availability

The-full length sequence of *NbCRVP* is available in GenBank under the accession number: OQ675540.
