# Supplementary material for: Non-transgenic, PAMAM co-delivery DNA of interactive proteins NbCRVP and NbCalB endows Nicotiana benthamiana with a stronger antiviral effect to RNA viruses
Source: J Nanobiotechnology. 2024 Jan 8;22:23. doi: 10.1186/s12951-023-02252-z (PMC10773047; doi:10.1186/s12951-023-02252-z)
Supplement: Supplementary file 1 — Additional file 1: Figure S1. The expression pattern of NbCRVP. (a) Expression of NbCRVP under the stress of viral infection at 1, 3, 5, 7 dpi. The data were analyzed by Duncan’s multiple range tests in the ANOVA program of SPSS, different letters indicate that values of the four treatments were significantly different (P < 0.05), which were the same as b, c, d. (b) The NbCRVP expression trends in flower, stem, leaf and root. (c) The NbCRVP expression trends after spraying with 0.5 mM SA, 0.05 mM ethephon, or 0.1 mM Me-JA, water with 0.02% Tween 20 was used as a control. (d) The expression of NbCRVP after silencing the key signaling genes, NPR1, COI1 and EIN2. (e) Subcellular distribution of NbCRVP observed in healthy N. benthamiana and virus infected N. benthamiana. Figure S2. Subcellular distribution of NbCRVP using DAPI as a nuclear localization marker. Figure S3. The silencing efficiency of NbCRVP. (a) Phenotype after 14 days of silencing NbCRVP. (b) The silencing efficiency was detected after 14 days of treatment. Figure S4 Overexpression NbCRVP on multi-infection of TMV, PVY and CMV. (a) The detection of NbCRVP after overexpression NbCRVP. The data were analyzed with independent sample t test, * indicated that values of the two treatments were significantly different (P < 0.05), the same as b. (b) Effect of overexpression NbCRVP on multi-infection of TMV, PVY and CMV detected by RT-qPCR at 1, 2, 3, 4 dpi. (c) Differences in biological symptoms of overexpression NbCRVP on multi-infection of TMV, PVY and CMV. Figure S5 Screening of interactive proteins for NbCRVP in yeast two-hybrid assay. (a) Screening of interacting proteins by dot culture on QDO/X/A. (b) PCR amplification, gel electrophoresis, and sequencing to identify interacting proteins. Figure S6 SEM and TEM images of PAMAM@CRVP nanocomposites. Figure S7 Detect the expression of PAMAM@CRVP:CalB in the Solanum lycopersicum and Capsicum annuum protoplast. (a) Detect the expression of PAMAM@CRVP:CalB in Sol [file 12951_2023_2252_MOESM1_ESM.pdf]

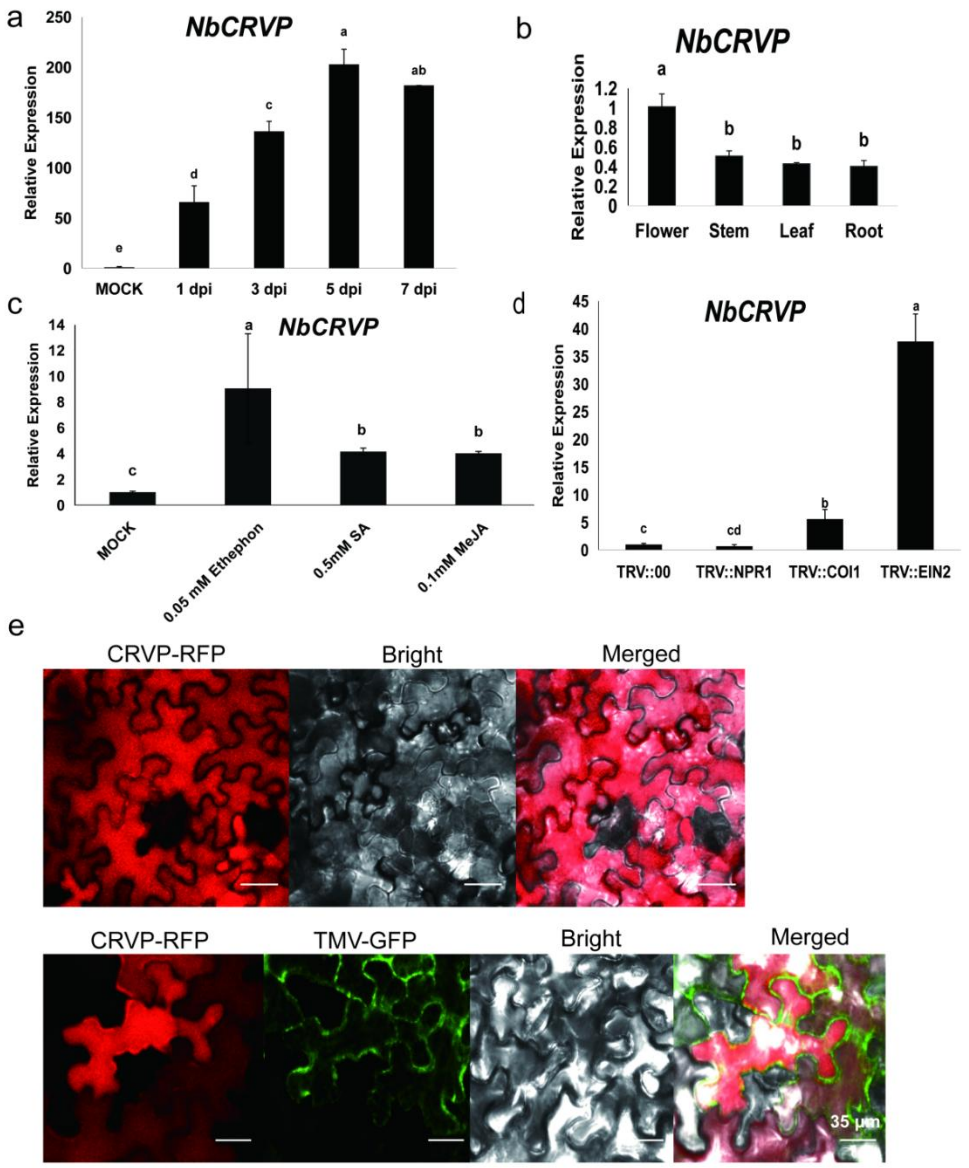

Figure S1 The expression pattern of *NbCRVP*. (a) *NbCRVP* expression under the stress of viral infection at 1, 3, 5, and 7 dpi. (b) *NbCRVP* expression trends in the flower, stem, leaf, and root tissues of *N. benthamiana*. (c) *NbCRVP* expression trends after spraying with 0.5 mM SA, 0.05 mM ethephon, 0.1 mM Me-JA, or 0.02% Tween 20 water solution, which was used as the control. (d) *NbCRVP* expression after silencing key signaling genes *NPR1*, *COI1* and *EIN2*. (e) Subcellular distribution of NbCRVP observed in healthy *N. benthamiana* and virus-infected *N. benthamiana*. Data shown in (a), (b), (c), and (d) were analyzed using Duncan's multiple range tests in the ANOVA program of SPSS, with different letters indicating significant differences among four different treatment groups ($P < 0.05$).

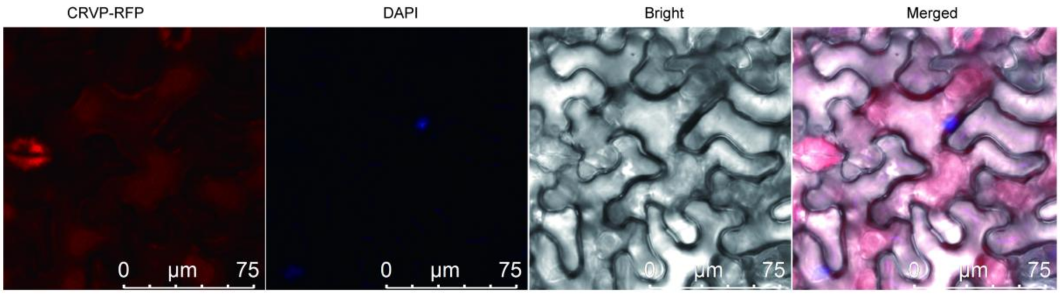

Figure S2 Subcellular distribution of NbCRVP using DAPI as a nuclear localization marker.

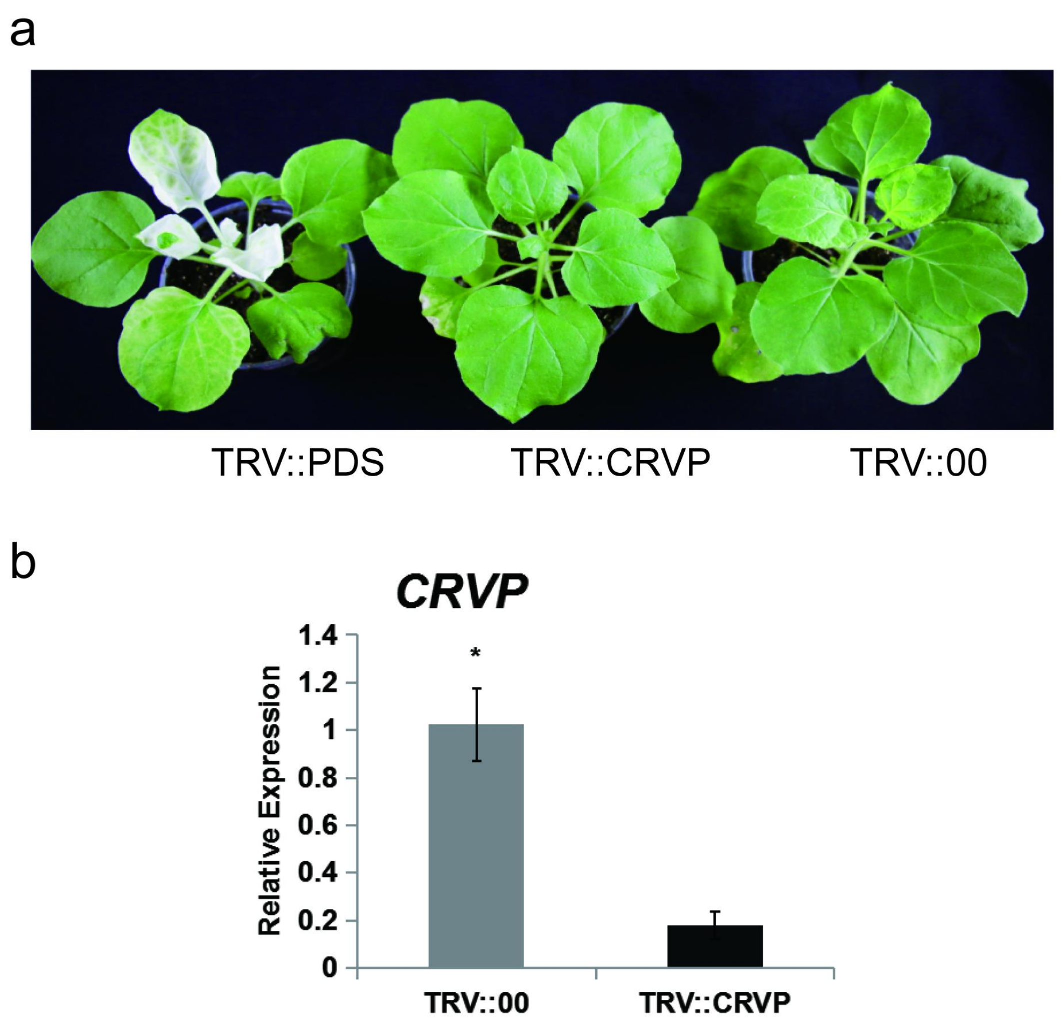

Figure S3 *NbCRVP* silencing efficiency. (a) Phenotype after 14 days of silencing *NbCRVP*. (b) *NbCRVP* silencing efficiency was detected after 14 days of treatment.

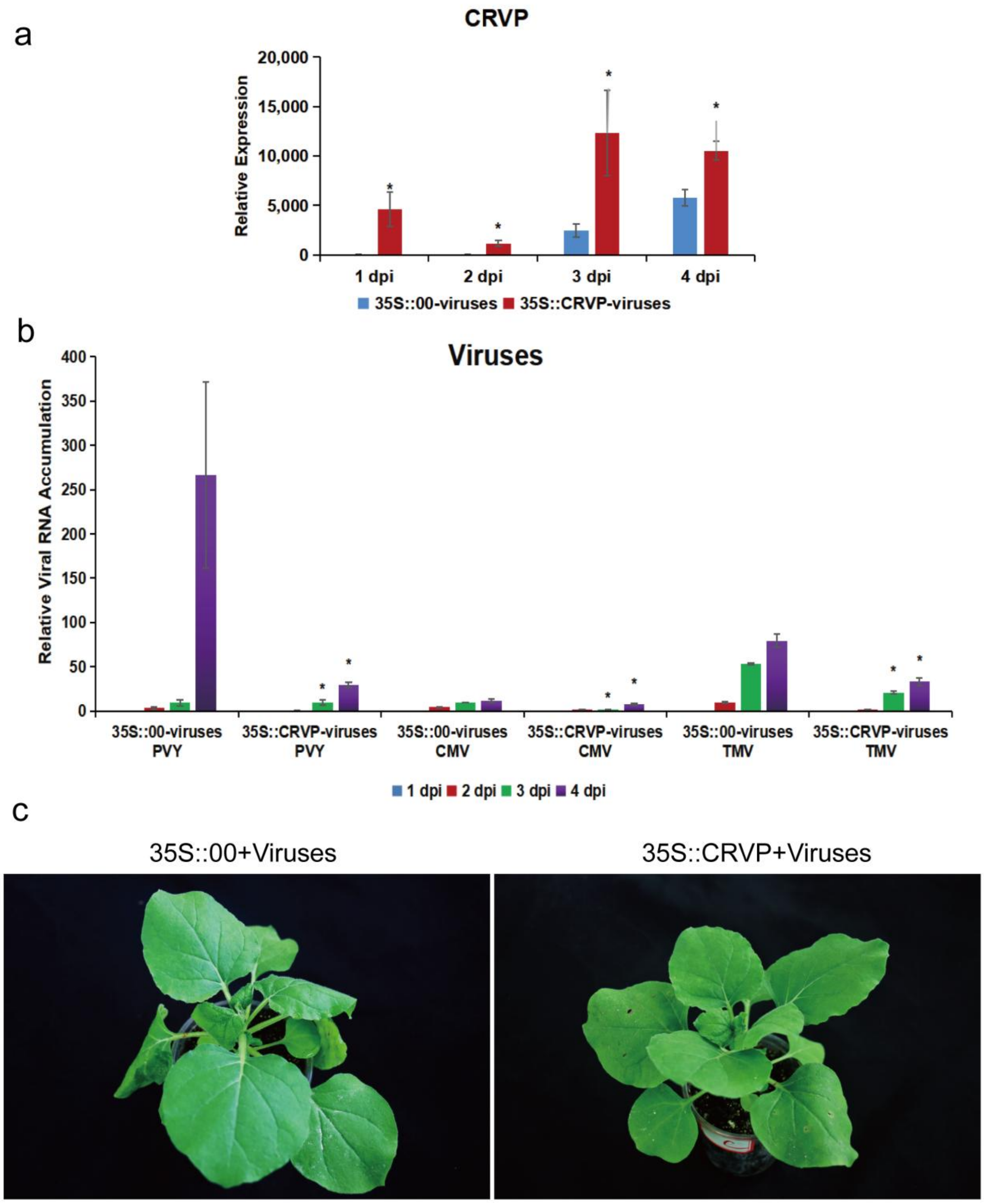

Figure S4 Effects of *NbCRVP* overexpression on multi-infection of TMV, PVY, and CMV. (a) *NbCRVP* expression levels after its overexpression. (b) Effect of *NbCRVP* overexpression on multi-infection of TMV, PVY, and CMV at 1, 2, 3, and 4 dpi, as detected by RT-qPCR. (c) Effect of *NbCRVP* overexpression on biological symptoms of TMV, PVY and CMV infection. Data shown in (a) and (b) were analyzed using the independent sample t test, with * indicating significant differences between two different groups ($P < 0.05$).

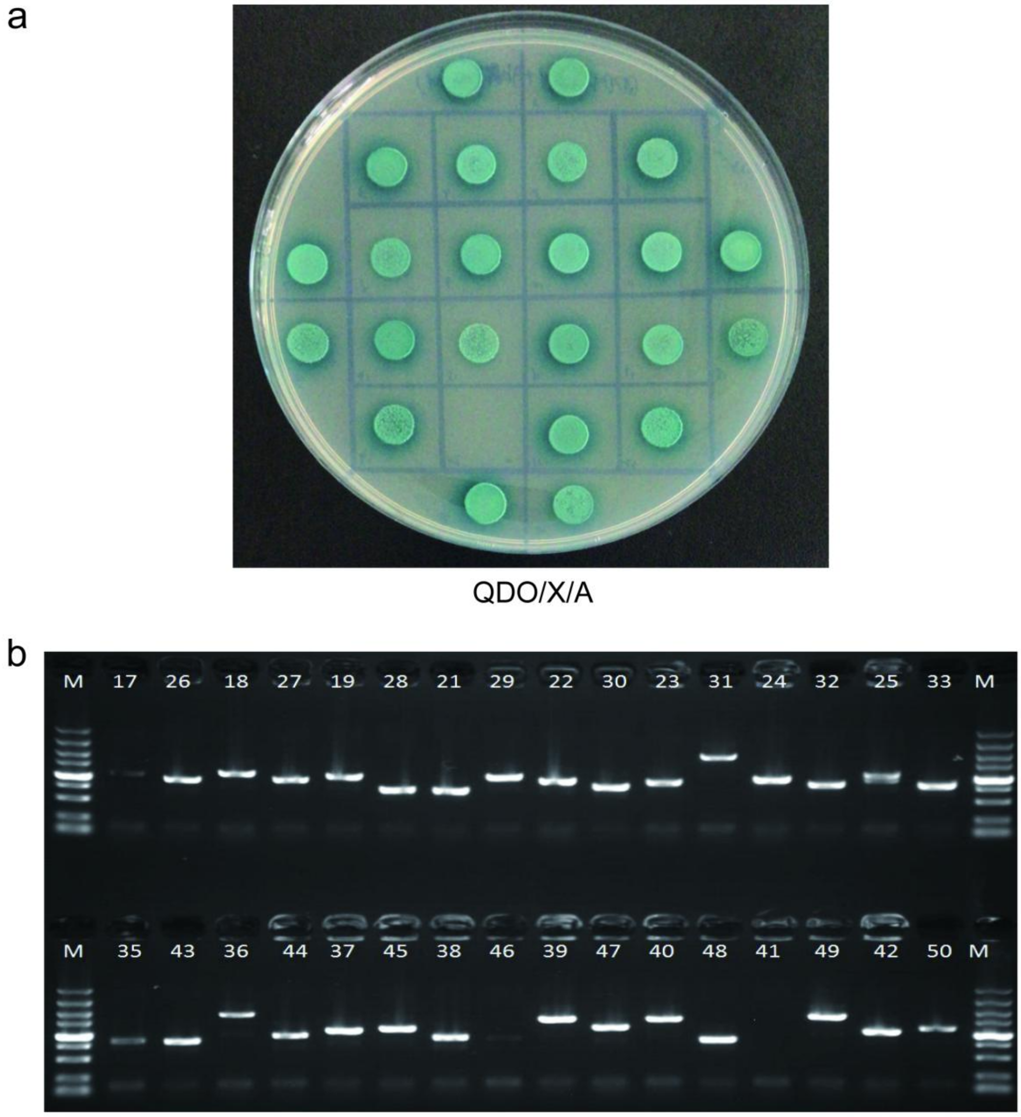

Figure S5 Screening of interactive proteins for NbCRVP by yeast two-hybrid assay. (a) Screening of interacting proteins for NbCRVP by dot culture on QDO/X/A. (b) PCR amplification, gel electrophoresis, and sequencing to identify NbCRVP-interacting proteins.

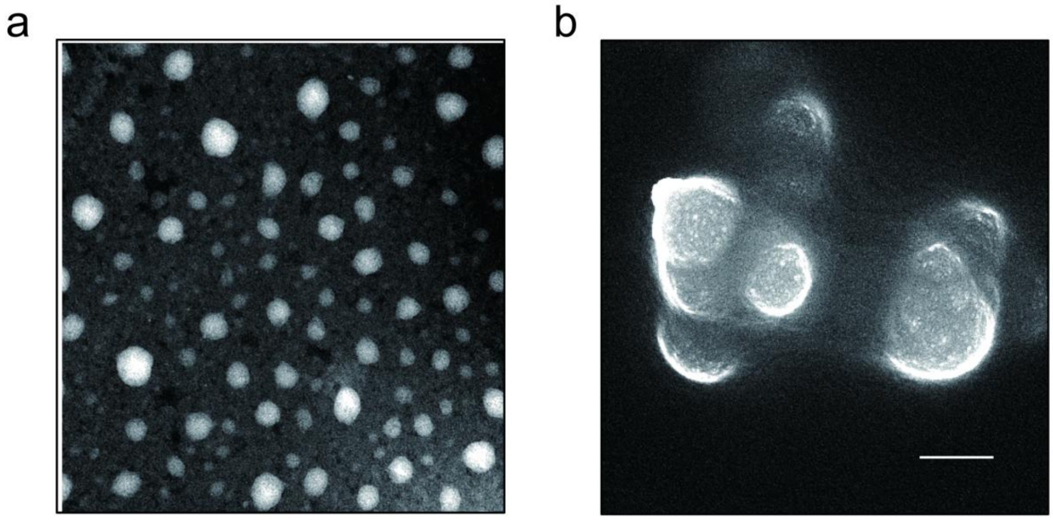

Figure S6 SEM and TEM images of PAMAM/CRVP nanocomposites.

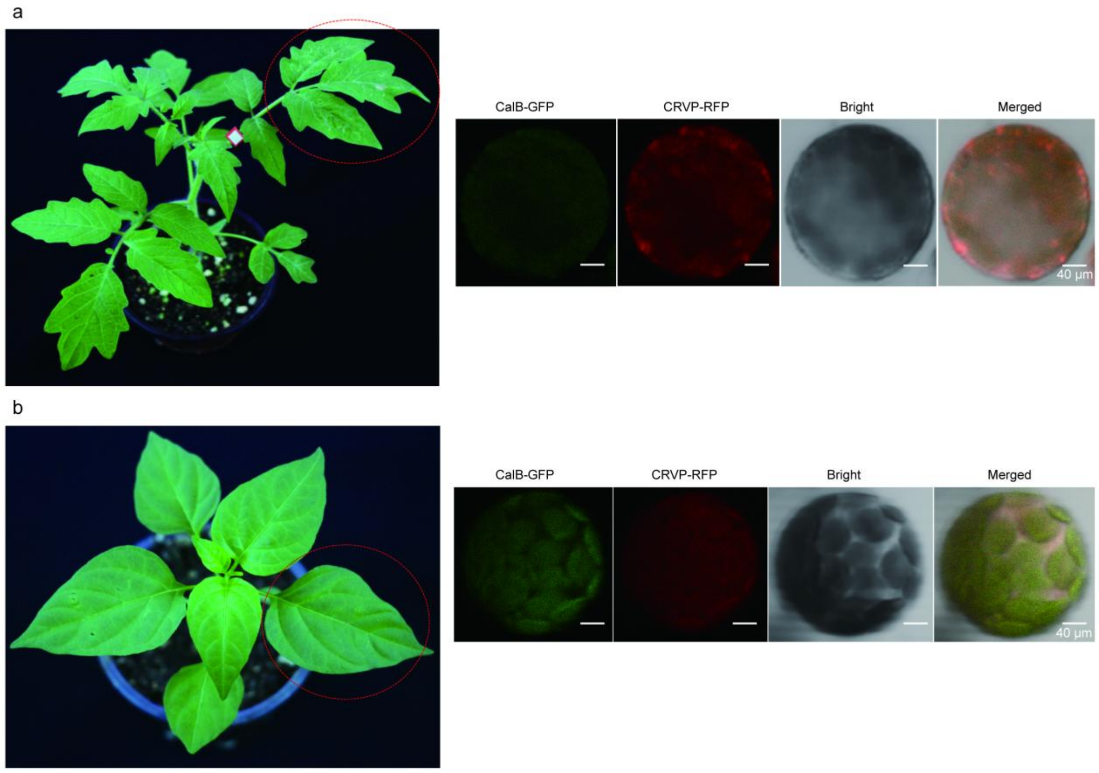

Figure S7 Detect the expression of PAMAM/CRVP:CalB in the *Solanum lycopersicum* and *Capsicum annuum* protoplast. (a) Detect the expression of PAMAM/CRVP:CalB in *Solanum lycopersicum.* (b) Detect the expression of PAMAM/CRVP:CalB in *Capsicum annuum.*

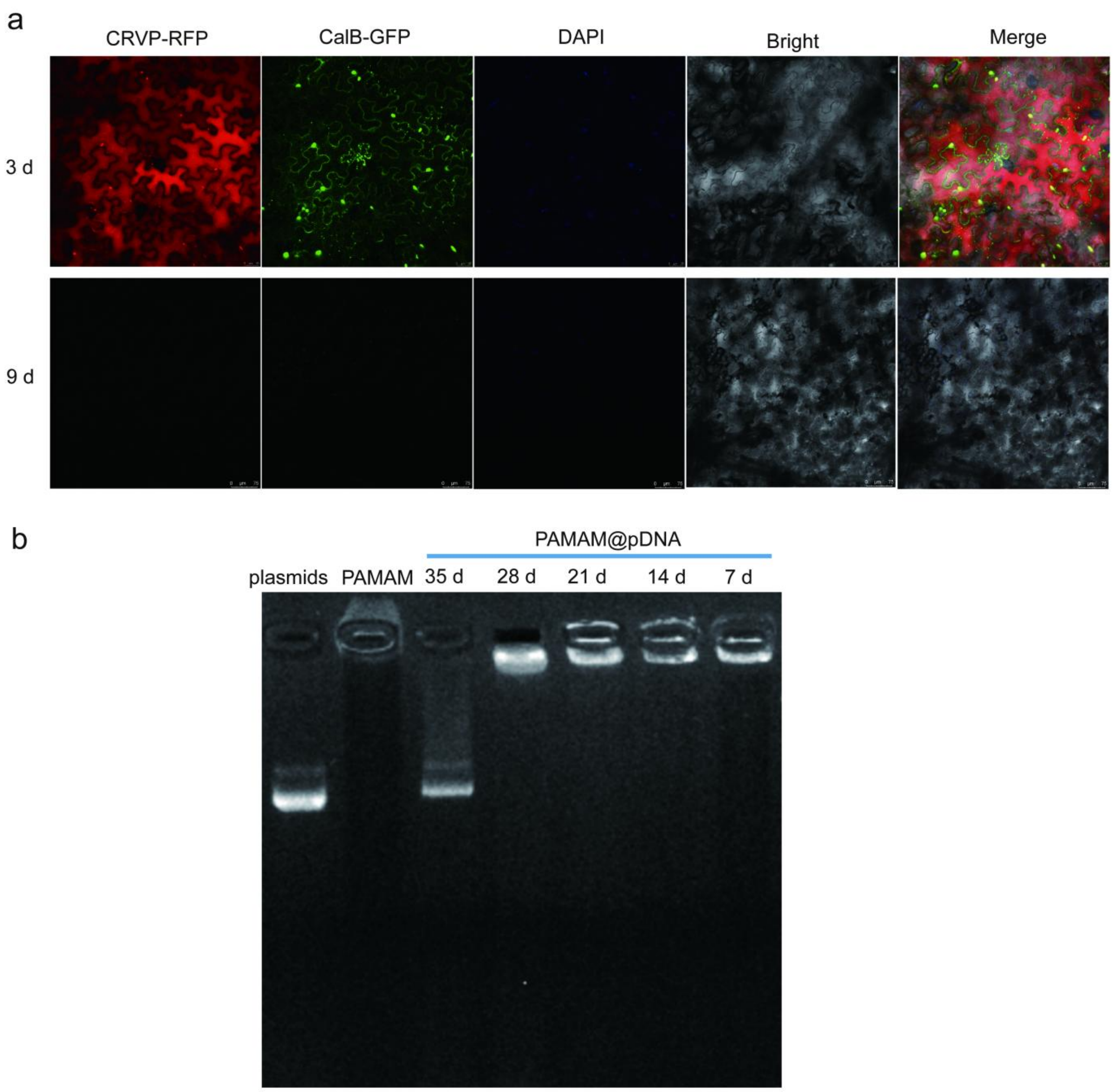

Figure S8 In vitro shelf life and delivery expression of PAMAM@pDNA nanocomplexes in plant cells. (a) Monitoring the delivery and expression of PAMAM@pDNA nanocomplex in plant cells under laser confocal. (b) Monitor the stability of PAMAM@pDNA nanocomplexes in vitro.

a

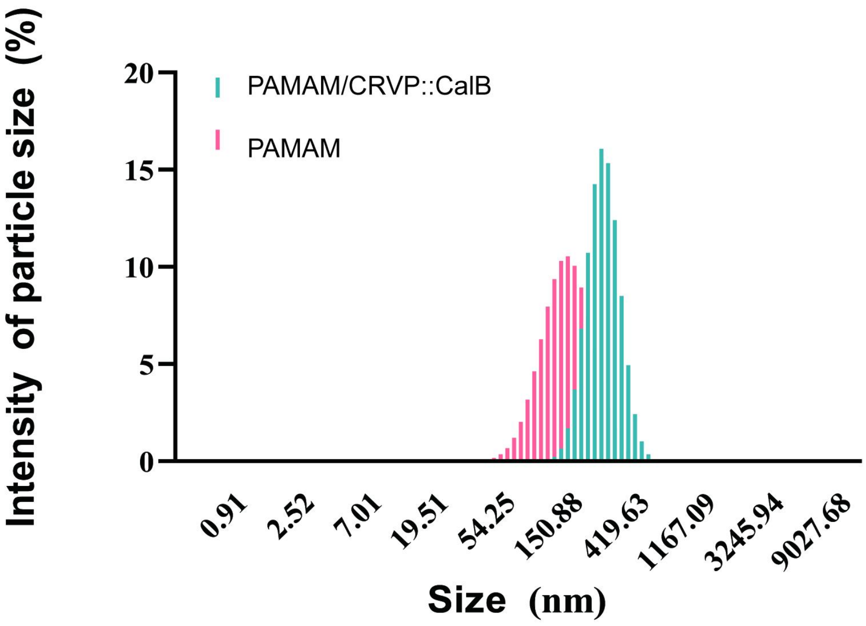

b

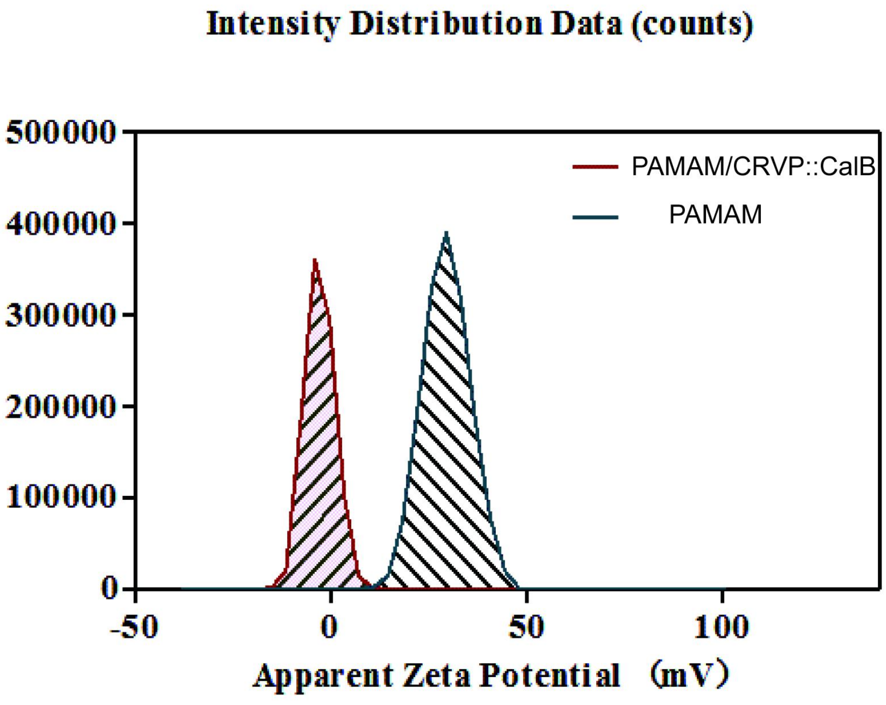

Figure S9 Characterization of PAMAM/CRVP:CalB nanocomposites. (a) Average particle size distribution of PAMAM/CRVP:CalB nanocomposites. (b) Intensity distribution of Zeta potential of PAMAM/CRVP:CalB nanocomposites.

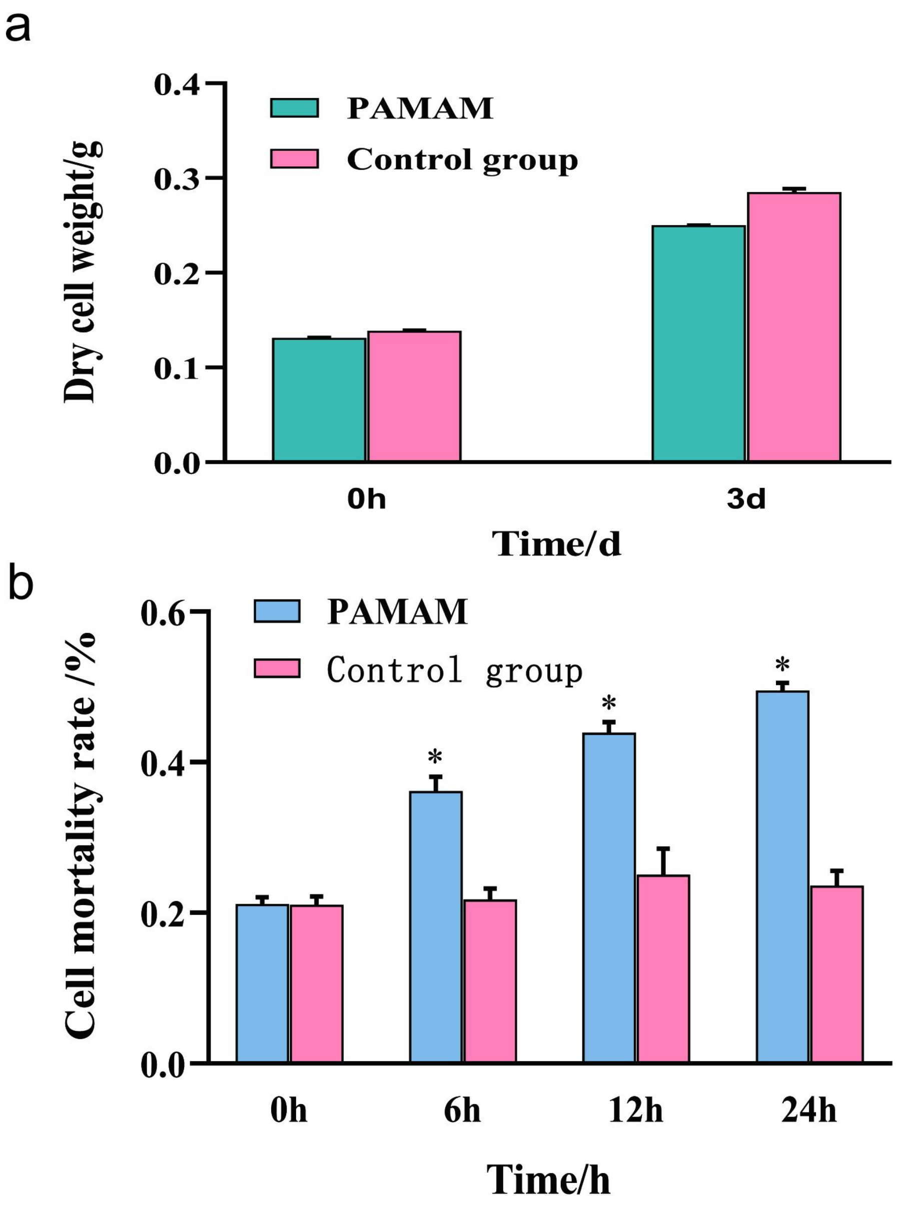

Figure S10 Toxicity assay of PAMAM/CRVP:CalB nanocomposites. (a) Dry cell weight determination treated with PAMAM/CRVP:CalB nanocomposites. (b) cell mortality rate determination treated with PAMAM/CRVP:CalB nanocomposites.

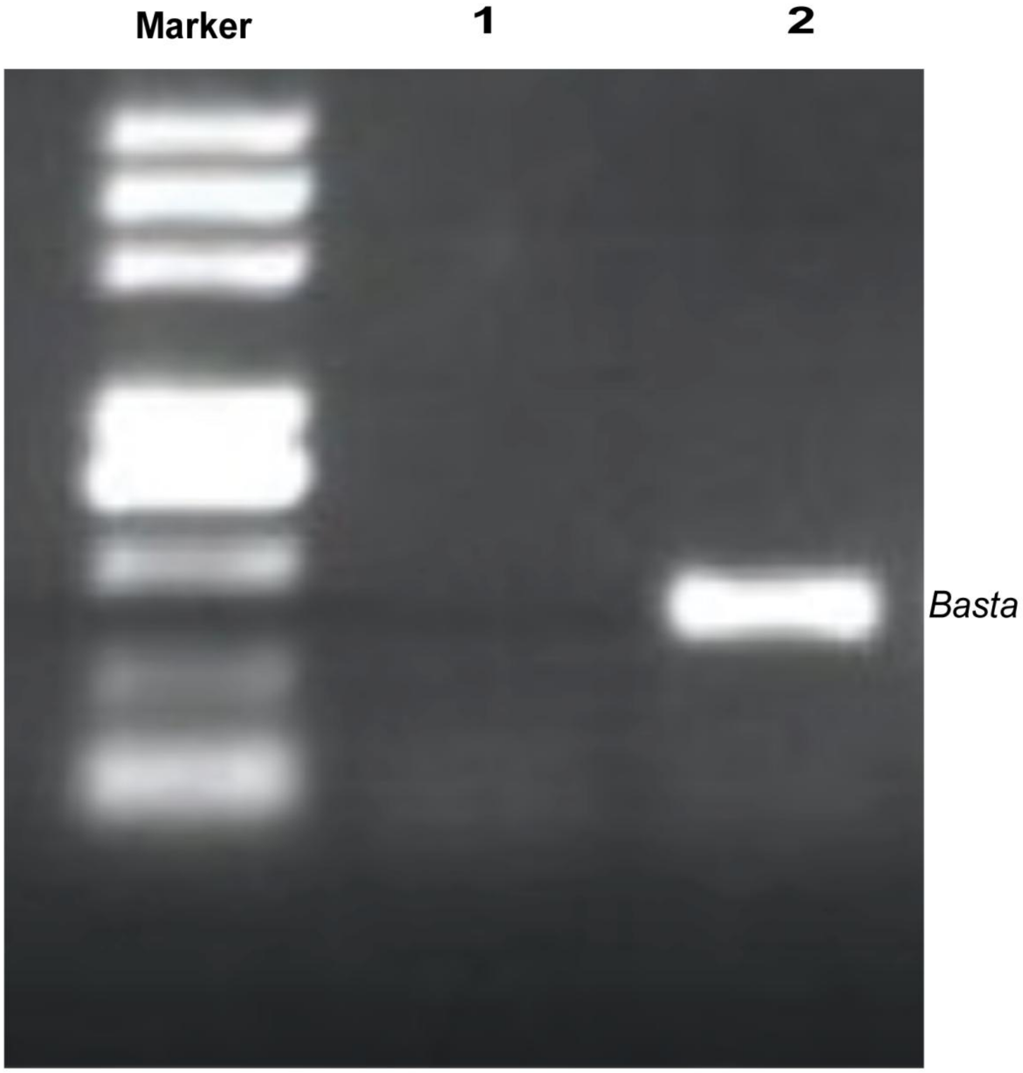

Figure S11 Validation that PAMAM-mediated resistance protein delivery was non-transgenic. Lane 1 was PAMAM@CRVP:CalB-treated wild-type *N. benthamiana* seeds, Lane 2 was PAMAM@CRVP:CalB-treated NbCRVP-OE seeds.
