# Supplementary material for: Non-transgenic, PAMAM co-delivery DNA of interactive proteins NbCRVP and NbCalB endows Nicotiana benthamiana with a stronger antiviral effect to RNA viruses
Source: J Nanobiotechnology. 2024 Jan 8;22:23. doi: 10.1186/s12951-023-02252-z (PMC10773047; doi:10.1186/s12951-023-02252-z)
Supplement: Supplementary file 2 — Additional file 2: Table S1. Primers used in this study. Table S2. Characterization data of PAMAM@CRVP nanocomposites. [file 12951_2023_2252_MOESM2_ESM.pdf]

Table S1 Primers used in this study

Primers	The sequence of primers
CRVP-F	5’-ATGGGATACTCCAAAACATTAGTTG-3’
CRVP-R	5’-GACATCAGTTGGAAGTTCCAACTTG-3’
CRVP QF	5’- CACCGGGAAAAGTGTGTGG-3’
CRVP QR	5’-CGTAGGGACGTTGTCCTCTC-3’
β-Actin QF	5’-CAAGGAAATCACCGCTTTGG-3’
β-Actin QR	5’-AAGGGATGCGAGGATGGA-3’
NPR1-TRVF	5’-TAAGGTTACCGAATTCATGGATAATAGTGGGACTGCGT -3’
NPR1-TRVR	5’-AGACGCGTGAGCTCGGTACCCGGACTCCTCGCCGACAA-3’
COI1-TRVF	5’-TAAGGTTACCGAATTCCTTGATAATGGTGTCCGTGC-3’
COI1-TRVR	5’-AGACGCGTGAGCTCGGTACCCCAACGTATCCCAGAAGCATC-3’
EIN2-TRVF	5’-TAAGGTTACCGAATTCATGGAATCTGAAACTCTGACTATAG-3’
EIN2-TRVR	5’-AGACGCGTGAGCTCGGTACCATATTCTTCACTGCAAAT CTGGGC-3’
NPR1 QF	5’-ATCTCTTGCTATGGCAGGCGATG -3’
NPR1 QR	5’-ACCGTTGTCCTCTGTGCGTTG -3’
COI1 QF	5’-GCTCCACGCGATTACCAACGG -3’
COI1 QR	5’-CTGCCACCATCTCTTGCACACC -3’
EIN2 QF	5’-GTATGGAATTCAGGAGCGGAAGGC -3’
EIN2 QR	5’-AGAAGACGGAAGCACAAGAGCAAC -3’
CalB-GFP-F	5’-CTTTAGATCTTCTAGAATGGCAACTGGTTCTTCCTC-3’
CalB-GFP-R	5’-CCATGAGCTCGAATTCATATTCAACACTGTAATTGCTGT-3’
BD-CRVP-F	5’-CATGGAGGCCGAATTCATGGGATACTCCAAAACATTAGTTG-3’
BD-CRVP-R	5’-GCCGCTGCAGGTCGACGACATCAGTTGGAAGTTCCAACTTG-3’
CRVP-RFP-F	5’-GGAGGCCAGTGAATTCATGGGATACTCCAAAACATTAGTTG-3’
CRVP-RFP-R	5’-TCATCTGCAGCTCGAGGACATCAGTTGGAAGTTCCAACTTG-3’
AD-CalB-F	GGAGGCCAGTGAATTCATGGCAACTGGTTCTTCCTC
AD-CalB-R	TCATCTGCAGCTCGAGCATATTCAACACTGTAATTGCTGT
TRV2-CRVP-F	5’-TAAGGTTACCGAATTCATGGGATACTCCAAAACATTAGTTG-3’
TRV2-CRVP-R	5’-AGACGCGTGAGCTCGGTACCAGCGGCGTTGAGTTGAGG-3’
E100F	5’-CATCATTGCGATAAAGGAAAGGC -3’
E100R	5’-GGCGGTAAGGATCTGAGCTACAC -3’
PVY-F	5’-GATTTGCCTAAGGGTTGGTTTCG -3’
PVY-R	5’- GATGAATGGGCTTATGGTTTGGTG-3’
TMV-F	5’-GAGTAGACGACGCAACGG -3’
TMV-R	5’-CCAGAGGTCCAAACCAAAC -3’
CMV-F	5’-GTGGGTGACAGTTCGTAAA -3’
CMV-R	5’-GTGGGAATGCGTTGGT -3’

Table S2 Characterization of PAMAM/CRVP nanocomposites.

PAMAM-NbCRVP (N/P ratio)	PAMAM (nmol)	NbCRVP (nmol)	Mean particle diameter (nm)	ζ potentia (mV)	Nanometer polydispersity index (DPI)
1:0	200	0	135.6±0.12	5.24±0.12	0.608
1:3	500	200	196.8±0.42	4.52±0.05	0.184
1:2	600	200	227.7±0.07	1.07±0.64	0.442
1:1	200	200	201.8±0.52	-2.83±0.95	0.140
2:1	66.67	200	263.7±0.12	-27.45±0.15	0.195
3:1	40	200	267.4±0.05	-34.35±0.25	0.436
